# Identification, characterization and control of a sequence variant in monoclonal antibody drug product: a case study

**DOI:** 10.1038/s41598-021-92338-1

**Published:** 2021-06-24

**Authors:** Anushikha Thakur, Rekha Nagpal, Avik Kumar Ghosh, Deepak Gadamshetty, Sirisha Nagapattinam, Malini Subbarao, Shreshtha Rakshit, Sneha Padiyar, Suma Sreenivas, Nagaraja Govindappa, Harish V. Pai, Ramakrishnan Melarkode Subbaraman

**Affiliations:** 1 Research and Development (RND), Biocon Biologics Limited, Biocon Park, SEZ, Plot No. 2&3,Phase-IV, Bommasandra Industrial Estate, Bommasandra-Jigani Link Road, Bangalore, 560099 India; 2 Divis Laboratories Limited, Chotuppal, Hyderabad, 508252 India; 3 Sunquest Information Systems, Bangalore, 560095 India; 4 Alvotech Hf, Saemundargata 15-19, 101 Reykjavik, Iceland

**Keywords:** Biophysics, Biotechnology

## Abstract

Sequence variants (SV) in protein bio therapeutics can be categorized as unwanted impurities and may raise serious concerns in efficacy and safety of the product. Early detection of specific sequence modifications, that can result in altered physicochemical and or biological properties, is therefore desirable in product manufacturing. Because of their low abundance, and finite resolving power of conventional analytical techniques, they are often overlooked in early drug development. Here, we present a case study where trace amount of a sequence variant is identified in a monoclonal antibody (mAb) based therapeutic protein by LC–MS/MS and the structural and functional features of the SV containing mAb is assessed using appropriate analytical techniques. Further, a very sensitive selected reaction monitoring (SRM) technique is developed to quantify the SV which revealed both prominent and inconspicuous nature of the variant in process chromatography. We present the extensive characterization of a sequence variant in protein biopharmaceutical and first report on control of sequence variants to < 0.05% in final drug product by utilizing SRM based mass spectrometry method during the purification steps.

## Introduction

Expressing the right clone is one of the important steps in the product development of protein biotherapeutics^1^. In spite of the near absolute fidelity of DNA polymerases, single nucleotide polymorphisms are observed due to erroneous gene transcription, which results in altered amino acid sequences. The sequence alterations can also result from mistranslation or improper tRNA acylation by either nonsense read-through or misreading at the level of transcription or translation^2^. Additionally, mis-cleavage during the posttranslational processing can also lead to non-native amino acid substitutions^3^. These sequence variants in the final drug product are undesirable, as they may possess altered physicochemical and or biological properties compared to wild-type product, which therefore can affect the overall efficacy, stability or safety of the biomolecule drug. The most unwanted outcome of these substitutions are the perturbations in tertiary structure of the protein leading to formation of new conformational epitopes which might elicit varying levels of unwanted immune responses. The safety consequences of immune responses to therapeutic protein products are generally unpredictable and can range from no apparent effect to serious adverse events depending on immune tolerance of the patient to that particular therapeutic protein. Recent survey conducted by International Consortium for Innovation & Quality in Pharmaceutical Development (IQ) demonstrated that biopharmaceutical industry has SV workflows incorporated in their early development with appropriate mitigation strategy to counteract specific mis-incorporation mechanisms at the genetic, translation, and cellular levels^4^. The survey also reported that several organizations discard cell lines with > 1% SV and understand that hard limits on SV is not practical and a cell line with SV can be used for further product development if adequate risk assessment for the criticality of its low abundant presence in the mAb drug product has been performed. The US Food and Drug Administration (US-FDA) guidelines recommends that the micro heterogeneity of pharmaceutical products that are not expected to change product performance should be characterized to ensure product consistency^5^. This means that, sequence variants if observed, their levels and control strategy need to be provided by the applicant at the time of registration. Thus, the detection of these sequence modifications early in product development is desirable.

The occurrence of amino acid substitution in a small population of the secreted protein, monoclonal antibodies in present context, has been reported by many biopharmaceutical companies in recent times^6–14^. It is important to understand the origin of sequence variants whether it is genetic, misincorporation or other modification to prevent their manifestation in the protein product. While genetic mutation is clone specific and appear at the mutation site, amino acid misincorporation can be found across entire protein sequence^10–15^. Biopharmaceutical industry consortium (IQ) reported occurrence 5–20% genomic mutations and 5–30% of mis-incorporations while analyzing multiple samples during early development^4^. Many technologies are available for detection of sequence modification at DNA/RNA and protein level. Real-time polymerase chain reaction (PCR) and mass spectrometry based methods are the more commonly used techniques for estimating the relative abundance of mutant species^4, 16–21^. However, in general the de-novo identification of these sequence modifications in clones are challenging due to their low abundance. Next-generation sequencing technologies (NGS) have been revolutionizing genome research by sequencing personal genomes, characterize genomic landscapes, and detect and identify a large number of low abundant sequence variants^19–21^. Advancements in NGS workflows have enabled detection of as low as 0.1% SV in production cell lines with 0.5% as reliable threshold^4,12^. NGS is now used as frontline to identify mammalian cell clones with genetic mutations. Zhang et.al. proposed used of NGS based identification (~ 0.2%) and mass spectrometry confirmation for cell line quality control on sequence variants throughout the different developmental stages^19^. The NGS leads can be used for more selective and targeted search of sequence substitutions in mass spectrometry (MS) based peptide mass fingerprinting analytics. Use of LC–MS/MS in SV workflow has been reported to confirm NGS identified low-level (0.4–1%) genetic SV in high-titer “top clones” of interest^21^.

The mass spectrometry based detection and identification of sequence variants is enhanced when coupled with UV based chromatographic separation techniques^8,22–26^. The altered physicochemical property of sequence variants may allow them to elute separately from the main variant in either ion exchange chromatography, hydrophilic interaction chromatography or reversed-phase chromatography. When present in considerable amounts, these new peaks can be identified and further characterized for any amino acid substitution(s) using mass spectrometry. Various factors that impact the quality of peptide map data include choice of enzyme, alkylating agent and duration of proteolytic cleavage for sample processing, resolution of mass analyzers, MS and MS/MS parameters with appropriate sensitivity and acquisition speed^18, 26, 27^. This improves SV detection by proteomic softwares. MS proteomic software like error tolerant search using MASCOT, MassAnalyszer (PepFinder™ and Biopharma Finder™ from Thermo-Fisher Scientific), Byologic/Byomap from ProteinMetrics and Expressionist from Genedata are commonly used for sequence variant detection using peptide mass fingerprinting data^3,17,28–37^. Identification of each peptide in software assisted MS detection is score based that is affected by the quality of MS/MS data which is again dependent on the abundance of the substitution, instrument sensitivity, degree of chromatographic separation, ionization efficiency of the separated peptides and ionization suppression by more dominant ions co-eluting in the complex matrix of the protein digest^38, 39^. In addition, these informatics tools often generate many false positives^3,15,40^ primarily due to misinterpretation of chemical modifications, N and C-terminal modifications as sequence variants. In addition, manual investigation, which is extensive and often time-consuming, is required to verify the data^18^. Recently published work from Wenzhou et al. reported use of PERL script to evaluate every identified hit to remove the false positives from the search results of PepFinder™^40^. Dynamic exclusion duration can also be used to reduce the effects of ionization suppression where repeated MS/MS scans of the most abundant precursor ions are disabled for specified time, thus allowing MS/MS detection of less abundant ions^41^. However, minimal influence of dynamic exclusion duration on the proteome coverage is also reported^27^. Many sequence variant containing peptides do not present exploitable physicochemical attribute(s) for chromatographic separation from wild-type sequences. Additionally, low-abundance peptides may not yield good MS/MS data for sequence identification with confidence. The detection is further limited by the speed with which the mass spectrometer can perform MS/MS experiments of ions that are observed in the survey scan. As a result, many sequence variants may escape detection at early clone screening and appear in later stages of product enrichment or scale-up productions. In such advanced stages where the product is characterized for its functional advances in efficacy, development of strategies to control sequence variant(s) in the desired product weighs over evaluating new clones.

High-resolution separation techniques and highly sensitive detection and quantitative methods are required for efficient control of the sequence variant(s). Yang et al. reported 0.03% as the limit of detection of well-resolved variant peptide relative to total peak area of all peptides in the tryptic peptide map in UV-PMF (ultra-violet detection based peptide mass fingerprinting) profile generated from linear ion trap quadrupole (LTQ)^3^. This corresponded to ∼3% (w/w) spiking of variant peptide containing antibody in control antibody. Similarly, 0.5% (w/w) was established as the limit of detection of PMF using extracted ion chromatogram. As low as 0.01% sequence variant was detected using LTQ Orbitrap by Yu et al. while the intermediate precision of 10–15% was established at 0.5%^11^. Post identification of the sequence variant, the quantitation limit, range, accuracy, and precision of any variant peptide are expected to be sequence dependent. More sensitive and selective methods like selected reaction monitoring (SRM) are required to perform quantitative analysis for very low abundant mis-incorporation events as part of routine product quality assessment^17^. Although most recent instruments are designed to perform this sophisticated analysis^39^, triple quadrupole mass spectrometers are most suited for this purpose due to the relatively higher selectivity.

Once a sequence variant is detected, the general approach is to reject the clone for further development to avoid adverse safety and efficacy related implications^4,21^. Genetic mutations cause more concern compared to mis-incorporations as change of cell line may be required while latter can be addressed by media optimization^4^. Depending on the stage of the development, this approach may incur a moderate to significant delay in reaching the drug to patients. Alternatively, the impact of very low levels sequence variant in a functionally inactive region of the protein can be nullified theoretically^9^ and the development can move forward. Although this approach avoids any delay in the program, it comes with a bigger risk- possibility of failing in the immunogenicity during the clinical trial. Here we report a third approach, where the physicochemical and the functional properties of a glutamic acid (E) to lysine (K) sequence variant, identified by LC–MS/MS in end of fermentation product during initial development of a monoclonal antibody based therapeutic, is studied thoroughly by an array of analytical techniques and additional process steps and highly sensitive analytical methods are implemented to make sure that the sequence variant containing version is efficiently controlled in the product. Multiple batches of drug products containing less than 0.04% sequence variant were thus manufactured using this approach. In this particular case, this approach not only avoided the delay due to starting again with a new clone but also mitigated the risk of failure in the clinical trial stage. Similar control strategy can be adopted for undesirable sequence variants using their unique physicochemical property.

## Results

### Early detection of the sequence variant

**Figure 1:**
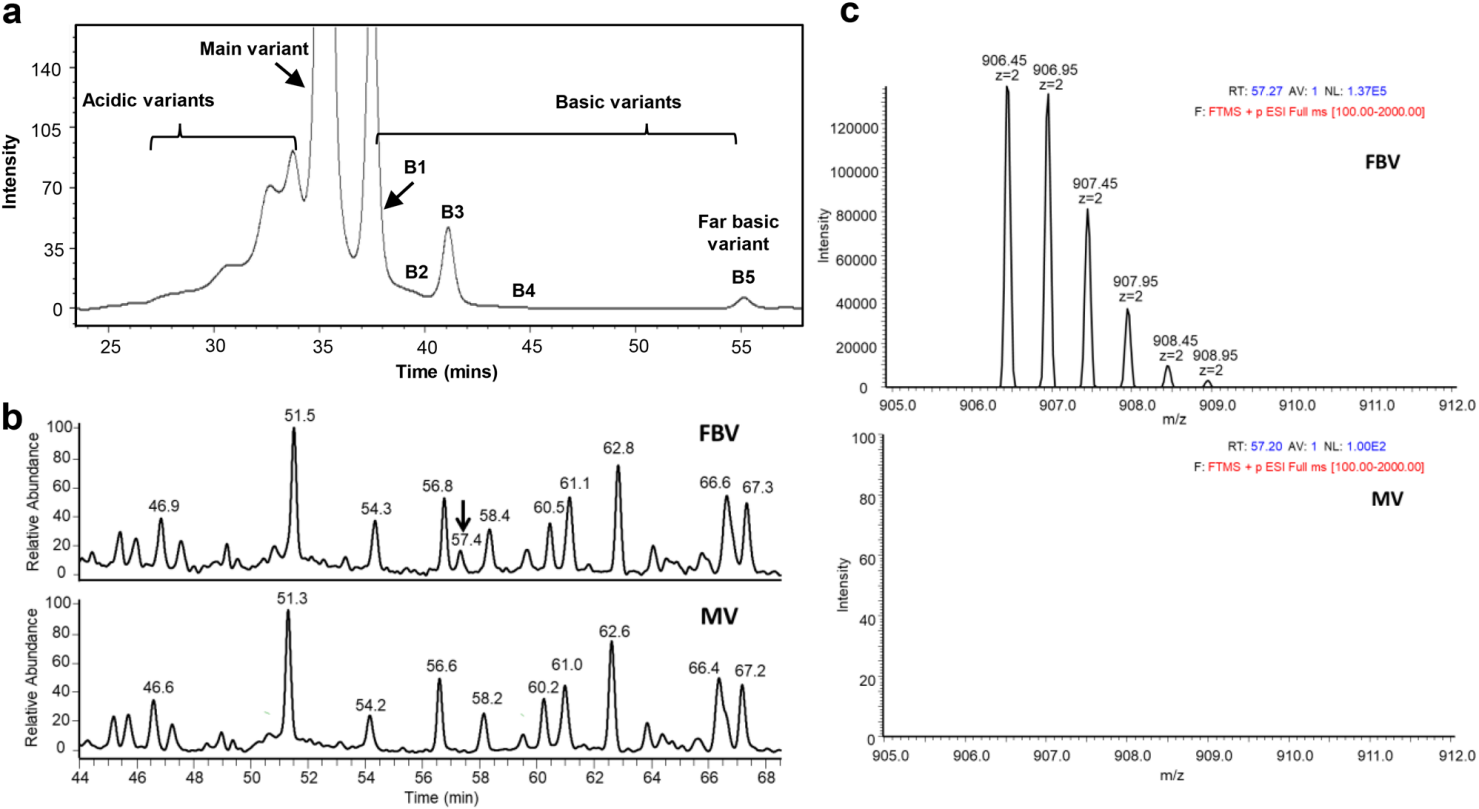
(**a**) Cation exchange (CEX) chromatogram of end of fermentation (EOF) product of mAb X. The acidic, main, basic (B1, B2, B3, B4) and far basic variant (B5) are marked. (**b**) Stacked overlay of a portion of tryptic peptide map UV profile of far basic variant (FBV, top panel) and main variant (MV, bottom panel) of mAb X. The extra signal (at RT = 57.39 min) in FBV profile is marked. (**c**) Comparison of MS signals observed around RT of 57–58 min in FBV (top panel) and MV (bottom panel). The m/z 906.45, z = 2 signal is observed in FBV and absent in MV.

The monoclonal antibodies (mAbs) undergo different chemical and enzymatic post-translational modifications (PTM). Although LC–MS/MS based peptide map analysis in high resolution mass spectrometers (HRMS) coupled with software driven search options during data analysis is a powerful tool to detect the PTMs and inherent modifications such as SVs, modifications present in very minute amounts (< 1%) may evade the software driven search due to the lack of definitive MS and/or MS/MS signals. Some of these modifications result in differences in the pI of the protein and subsequently lead to the acidic (lower pI) and basic (higher pI) variants of the mAb. These charge variants are separated by ion-exchange chromatography (IEX) and characterized to understand the nature of the PTM. The probability of identifying the PTMs and other variants are enhanced in the purified charged variants due to the enrichment of the modifications in these fractions. The Glutamic acid (E) to Lysine (K) sequence variant described here was first identified during the charge variant characterization of a far basic variant (FBV) in the end-of-fermentation product (EOF) of a monoclonal antibody (mAb X) (Fig. 1a). The protein A purified mAb X was fractionated through CEX and fractions enriched in FBV and the main variant (MV) was analyzed side by side extensively by mass spectrometry to understand the modification present in FBV.

The intact and sub-unit (heavy chain and light chain) mass of the charge variant FBV are compared with the main variant (MV) in Table S1 (supplementary material). The main variant deconvolutes to an intact mass of 148,082 Da comprising of two light and two heavy chains with dominant glycoform G0F (termed as G0F/G0F). Additionally, trace amounts of other glycoforms (G1F and G2F) were also observed. The same species were identified in FBV. Lysine (K) variant (addition of 128 Da for one K), due to the incomplete processing of C-terminal lysine on both the heavy chains of mAb X resulting in a mass of 148,337 Da (termed as G0F/G0F + 2 K), was also observed in FBV and no additional species was identified. Similarly, in reduced mass analysis, a mass of 50,646 Da corresponding to the dominant G0F isoform of heavy chain, was observed both in main variant and FBV, while an additional mass of 50,775 Da was observed in FBV, indicating C-terminal lysine variant (G0F + K) of single heavy chain. The mass of light chain observed in main variant and FBV was comparable to the theoretical mass of 23,412 Da and no additional mass was observed in FBV.

C-terminal lysine variants are known modifications in monoclonal antibodies that add positive charge to the net surface charge of the molecule imparting basic nature to the antibody^42^. The extracellular carboxypeptidase in mammalian expression systems generally clips off the C-terminal lysine at the heavy chain, the unprocessed anti-bodies appear as basic variants in the purified pool and add to antibody heterogeneity^43^. However, in mAb X cation exchange profile, the lysine variants (G0F/G0F + K and G0F/G0F + 2 K) elutes just after the main variant (peaks B1, B2, B3) and much before the far basic variant B5 (Fig. 1a). Thus, the far basic nature of charge variant FBV cannot be explained by the presence of lysine at the C terminus of HC alone and therefore needed further investigation.

Peptide mass fingerprinting (PMF) is a powerful technique for characterizing the primary structure of proteins including its amino acid sequence and posttranslational modifications (PTMs)^44^. For complete sequence coverage, complementary enzymes are used to generate peptides, which can be separated on reversed phase-high or ultra-performance liquid chromatography (RP HPLC/UPLC) and detected with UV detector^45^. The separated peptides are then investigated for amino acid sequence and PTMs using an accurate, high-resolution and sensitive mass spectrometer.

**Figure 2:**
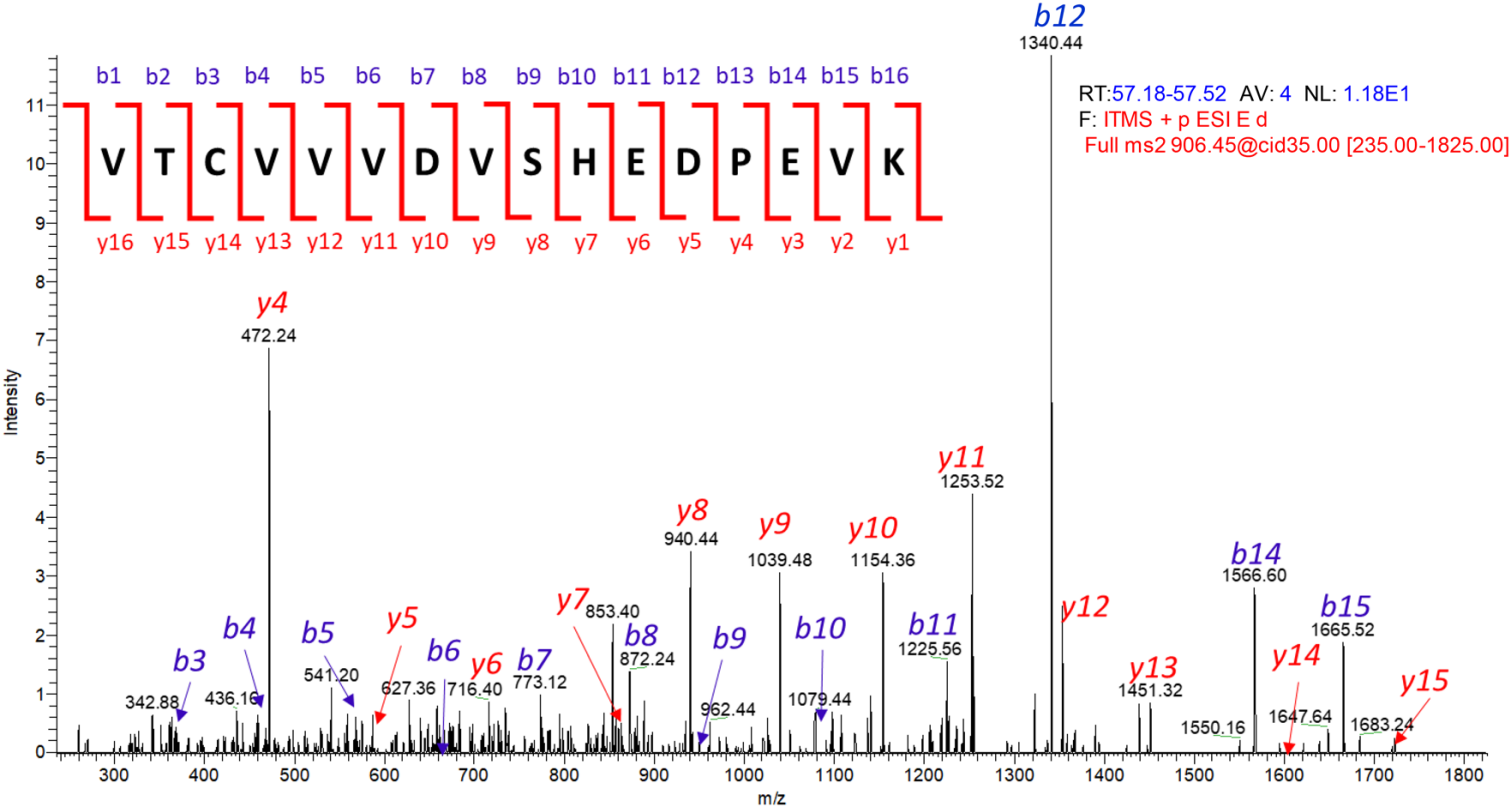
MS/MS analysis of the extra signal at m/z 906.45 (z = 2) elucidating the amino acid sequence.

**Figure 3:**
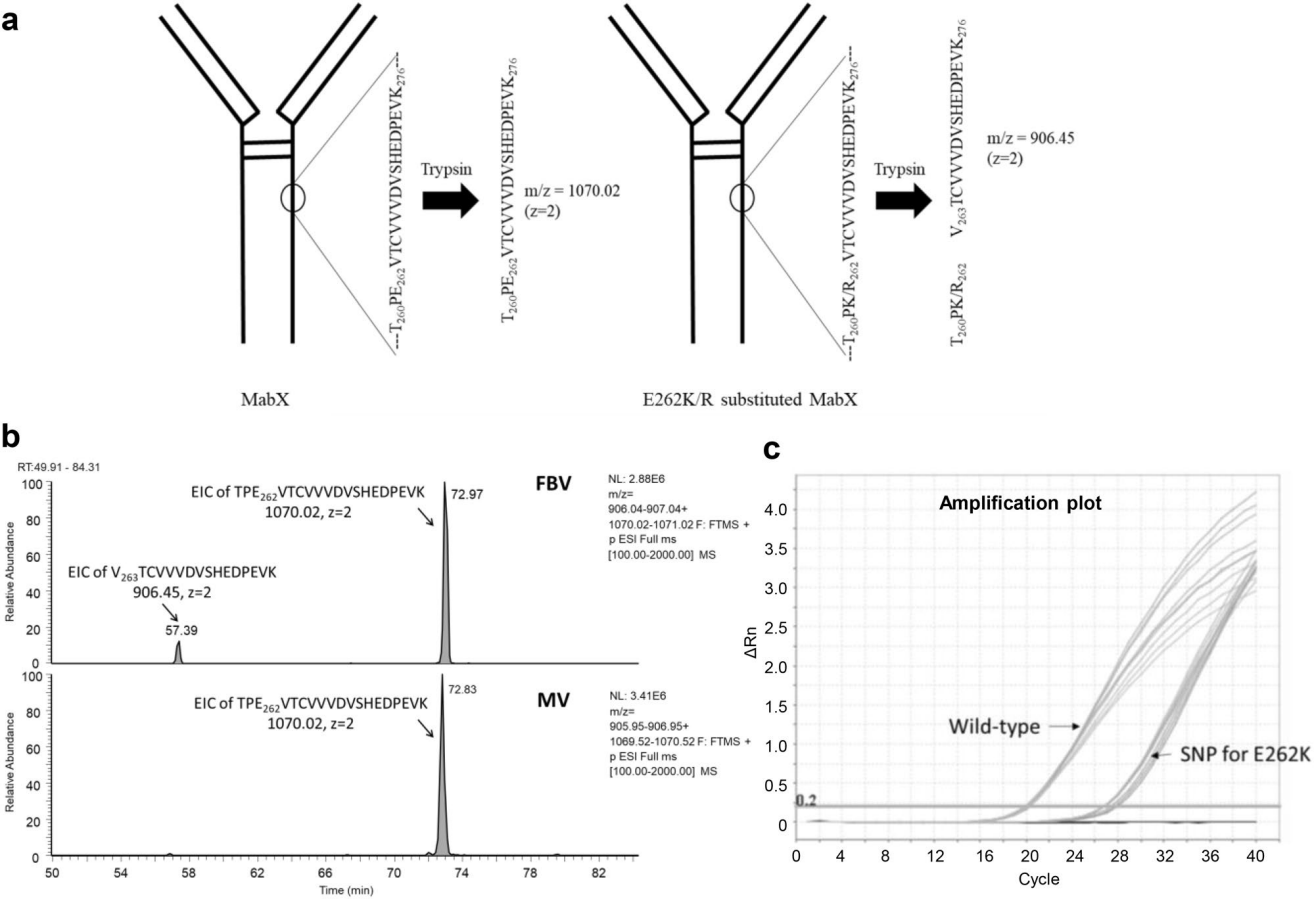
(**a**) Schematic of generation of truncated tryptic peptide V_263_TCVVVDVSHEDPEVK from substituted peptide TPK/R_262_VTCVVVDVSHEDPEVK in mAb X. (**b**) Extracted ion chromatogram of native peptide TPE_262_VTCVVVDVSHEDPEVK and truncated peptide V_263_TCVVVDVSHEDPEVK in FBV and MV of mAb X. Signals from both the peptides are visible in FBV, while only the native peptide is present in MV. (**c**) PCR amplification plot showing the amplification of primers containing the wild-type sequence and with the sequence containing the SNP corresponding to the E262K SV.

The enriched main and far basic variants of mAb X were digested by trypsin and the peptides thus generated was separated by liquid chromatography (LC) using a 120 min long gradient of 0.09% TFA in 90:10 acetonitrile: water. The separated peptides were detected by UV detector and then identified by mass spectrometer (Orbitrap LTQ) coupled to the LC outlet. Figure 1b presents a part of the PMF-UV profile overlay of mAb X charge variants MV and FBV. The UV profile overlay was comparable for all the peaks observed except an extra signal observed at 57.39 min in FBV (Fig. 1b). The mass spectrometry (MS) profile of this extra UV signal revealed monoisotopic mass at m/z 906.45 (z = 2), which was not present in MV (Fig. 1c). The single charged (z = 1) m/z of 1811.88 was also observed, however, z = 2 was the dominant charge state. Furthermore, MS/MS analysis identified the sequence of the peptide as VTCVVVDVSHEDPEVK (Fig. 2). This peptide appeared to be truncated part of the heavy chain tryptic peptide TPE_262_VTCVVVDVSHEDPEVK_278_ eluting at ~ 72 min (m/z 1070.02, z = 2) in both FBV and MV (refer Fig. 3b). Since the amino acid preceding V_263_ is E_262_, trypsin should not cleave at that site. One possibility is that the FBV contains a shorter version of the mAb X, truncated at E_262_. Truncation at heavy chain E_262_ site of mAb X will result in a protein with mass of 22,759 Da (with G0F mass), which could be easily detected by intact and sub-unit mass analysis. Further, the fragmented protein will be detected by other impurity identification techniques such as size exclusion chromatography (SEC) or CE-SDS. However, the truncated protein was not identified in FBV by intact and reduced mass analysis (Table S1) and by SEC or CE-SDS analysis (data not shown). This led to the hypothesis that, some population of the secreted mAb X is expressing K or R at the 262 amino acid position of heavy chain, instead of E, and thus presenting an additional cleavage site for trypsin, subsequently resulting in a shorter peptide V_263_TCVVVDVSHEDPEVK_278_ instead of the expected peptide T_260_PE_262_VTCVVVDVSHEDPEVK_278_ (Fig. 3a) during trypsin digested peptide map analysis. However, the other part of the peptide (T_260_PK_262_ or T_260_PR_262_) was not detected in this experiment, mostly because of the small size of it. Thus, the actual substitution (E to K or E to R) could not be confirmed from this experiment. Nevertheless, E to R substitution would lead to a mass difference of 27 Da in heavy chain mass, which can be detected by intact and reduced mass analysis. On the other hand, E to K substitution would lead to only 1 Da of mass difference and is not expected to be detected by intact and reduced mass analysis. Thus, no mass difference (apart from lysine variants) observed in FBV during intact and sub-unit mass analysis (Table S1) indirectly indicates the presence of E262K substitution. This hypothesis was further verified by Glu-C digested peptide map, as described below.

The extracted ion chromatograms of native peptide T_260_PE_262_VTCVVVDVSHEDPEVK_278_ and truncated peptide V_263_TCVVVDVSHEDPEVK_278_ in FBV and MV are shown in Fig. 3b, indicating the presence of both the peptides in FBV and only the native peptide in MV. The partial purity of FBV could lead to the presence of wild-type mAb X in FBV, subsequently generating the native peptide. Additionally, it is also plausible that E262K/R mutation is present only in one heavy chain of the E262K/R substituted mAb X, thus generating both native and substituted peptides during the PMF analysis of FBV. The presence of this truncated peptide was searched by extracted ion chromatogram in all the enriched charge variants of mAb X and it was found to be unique to FBV.

The E262K/R substitution was further confirmed using Glu-C enzymatic digestion of enriched FBV. Fig. S1a shows schematic of Glu-C digested wild-type and substituted mAb X. The native sequence L_237_LGGPSVFLFPPKPKDTLMISRTPE_262_VTCVVVDVSHEDPE_276_ would generate L_237_LGGPSVFLFPPKPKDTLMISRTPE_262_ and V_263_TCVVVDVSHEDPE_276_ as fragments post Glu-C digestion (in bicarbonate buffer), while the E262K or E262R substituted peptide would not undergo digestion at 262 site and appear as L_237_LGGPSVFLFPPKPKDTLMISRTPK_262_VTCVVVDVSHEDPE_276_ or L_237_LGGPSVFLFPPKPKDTLMISRTPR_262_VTCVVVDVSHEDPE_276_. The masses corresponding to these peptides were searched, through extracted ion chromatogram (EIC), in the mass spectrometry data from the Glu-C digested peptide map of main variant and far basic variant of mAb X. Among these, the mass corresponding to peptides L_237_LGGPSVFLFPPKPKDTLMISRTPE_262_ and V_263_TCVVVDVSHEDPE_276_ was observed in MV and FBV, while the mass corresponding to L_237_LGGPSVFLFPPKPKDTLMISRTPK_262_VTCVVVDVSHEDPE_276_ was detected in FBV (m/z = 1435.75, z = 3 and m/z = 1077.07, z = 4) (Fig. S1b) only. Peptide L_237_LGGPSVFLFPPKPKDTLMISRTPR_262_VTCVVVDVSH- -EDPE_276_ (m/z = 1445.09, z = 3 and m/z = 1084.07, z = 4) was not detected in any of these samples. The signal corresponding to L_237_LGGPSVFLFPPKPKDTLMISRTPK_262_VTCVVVDVSHEDPE_276_ (m/z = 1435.75, z = 3 and m/z = 1077.07, z = 4) was distinguished from the trace amounts of undigested L_237_LGGPSVFLFPPKPKDTLMISRTPE_262_VTCVVVDVSHEDPE_276_ (m/z = 1436.07, z = 3 and m/z = 1077.30, z = 4) present in FBV sample by the difference in monoisotopic mass obtained from the high resolution Orbitrap mass spectrometer. Presence of this peptide was further confirmed by the MS/MS analysis (Fig. S1c). Taken together, trypsin and Glu-C digested peptide map MS & MS/MS analysis of MV and FBV confirms the presence of E262K substituted mAb X in FBV. Substitution of an acidic amino acid (glutamic acid) to a basic amino acid (lysine) also explains the basic nature of the E262K substituted mAb X.

### Origin of the E to K substitution

Single nucleotide polymorphism (SNP) in the genomic DNA is one of the most common origin of sequence variant in the resultant protein^7,28^. In order to detect the SNP at the genomic level leading to E262K substitution, Cast-PCR (Competitive allele-specific Taqman qPCR) technique^46^ was employed. The technique utilizes an allele specific primer for somatic mutant allele detection that competes with an MGB blocker oligonucleotide to suppress the predominant wild-type background thus allowing 1:1000 (mutant: wild type allele) sensitivity. The amino acid E262K is possible only when the triplet codon ‘**g**ag’ changed to ‘**a**ag’ and therefore, primers were designed accordingly. In brief, the genomic DNA extracted from mAb X clone was analyzed by qPCR using primers specific to wild type (atgatctcccggacccct**g**aggtcacatgcgtggtggtggacgtg) and primer specific to sequence variant (atgatctcccggacccct**a**aggtcacatgcgtggtggtggacgtg). The amplification was observed using both the primers (Fig. 3c), indicating the presence of the SNP specific to base change from ‘g’ to ‘a’ which lead to E262K at protein level. Moreover, the relative abundance of the SNP was estimated from the cycle threshold (Ct) of the PCR reactions and was found to be ~ 1%. The presence of this SNP was further confirmed through next generation sequencing (NGS) by using both Illumina and Ion-Torrent platforms (data not shown).

### Characterization of the SV containing mAb X (mAb X’)

The structural and functional features of the modified (E to K substituted at 262 position in heavy chain) mAb X was assessed by several physicochemical and in-vitro bioassay techniques. This study was conducted to understand the structural and functional differences in the SV containing mAb X (called as mAb X’ from here on), compared to the native mAb X. Different lots of mAb X may have small differences in product related variants, due to the complex process involved in mAb manufacturing. Also, the inherent variabilities present in the analytical techniques used may also lead to small differences in the variant contents in different lots of mAb X. Thus, data from multiple lots of mAb X (manufactured in-house and sourced from external agencies) was utilized to obtain a range of data for mAb X and the data generated for mAb X’ was compared against that range. Nevertheless, to assess the presence of new impurities/variants or to understand the profile differences in case of peptide map and higher order structure methods, three mAb X lots were analyzed side by side with the mAb X’ lot.

**Figure 4:**
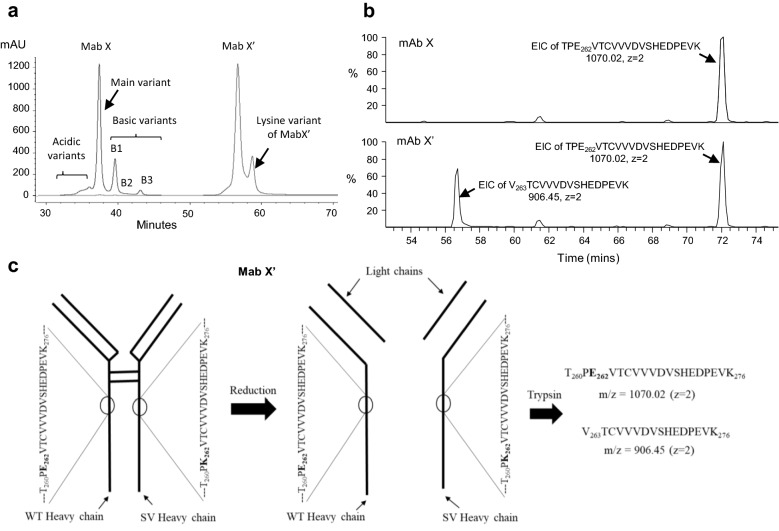
(**a**) Overlay of CEX profiles of mAb X and purified E262K containing mAb X (mAb X’). (**b**) EIC of the truncated peptide (E262K peptide) V_263_TCVVVDVSHEDPEVK_278_ and native peptide TPE_262_VTCVVVDVSHEDPEVK_278_ in mAb X’ (upper panel) and mAb X (bottom panel). EIC signal corresponding to truncated peptide and native peptide is marked in mAb X’ chromatogram while only the EIC signal corresponding to native peptide was detected and marked in mAb X chromatogram. (**c**) Schematic showing the generation of native and truncated peptide by tryptic digestion of mAb X’.

The mAb X’ was enriched and purified from mAb X by cation exchange chromatography and the purity (~ 98%) was confirmed by analytical cation exchange chromatography (Fig. 4a). The second peak observed in purified mAb X’ was found to be lysine variant (discussed below). Post purification, mAb X’ was buffer exchanged to the mAbX formulation buffer and stored appropriately.

The results obtained from the characterization of mAb X’ is summarized in Table 1. The primary structure of the mAb X’ and mAb X was compared by intact and sub-unit mass analysis and amino acid sequencing by LC–MS. The intact mass of mAb X’ and mAb X was similar and same heavy chain and light chain mass was observed for these two proteins as well (Table S1, Supplementary material). Apart from the extra tryptic peptide (V_263_TCVVVDVSHEDPEVK_278_) due to the E262K substitution in mAb X’, no other difference was detected in the amino acid sequence of mAb X’ and mAb X. Although the mAb X’ was not contaminated with mAb X (Fig. 4a), a significant amount of native peptide (T_260_PE_262_VTCVVVDVSHEDPEVK_278_) was detected in the tryptic peptide map mAb X’ (Fig. 4b). This indicates that the E262K substitution is present in only one of the heavy chains of mAb X’, while the other chain is unmodified. Thus, during reduction, mAb X’ generates equal amounts of native and modified heavy chains (Fig. 4c) and produces almost equal amounts of native and truncated peptides, post trypsin digestion.

**Table 1:** Summary of comparative characterization of mAb X’. (n = number of mAb X lots analysed to obtain the mAb X range).

Attribute	mAb X range	mAb X’	Analytical technique
**Intact mass (Da) (n = 6)**			
(G0F/G0F)	148,082–148,086	148,084	LC–MS
**Reduced mass (Da) (n = 3)**			
Light chain	23,410–23,413	23,412	LC–MS
Heavy chain (G0F)	50,640–50,645	50,646	
Amino acid sequence (n = 3)	The amino acid sequence of mAb X and mAb X’ was identical except the presence of E262K SV in mAb X’		Reduced peptide map LC–MS/MS
Disulfide map (n = 3)			Non-reduced peptide map
	All the expected disulfide linkages are intact in mAb X and mAb X’. Extra peptide observed in mAb X’ profile due to the E262K SV		LC–MS
Secondary structure (n = 3)	mAb X’ profile similar to the mAb X profile		Far-UV CD
**Secondary structure (n = 6)**			
β-sheet (%)	52–58	54	FTIR
β-turn (%)	19–22	21	
Random (%)	23–26	25	
Tertiary structure (n = 3)	mAb X’ profile similar to the mAb X profile		Near-UV CD
**Thermal unfolding (n = 3)**			
Tm1 (°C)	66–68	67	DSC
Tm2 (°C)	72–74	74	
Tm3 (°C)	82–83	84	
**Aggregate content (n = 10)**			
High molecular weight species (%)	0.2–0.3	0.1	SE-HPLC
**Fragment content (n = 10)**			
Total fragments (%)	1.8–2.2	1.9	NR CE-SDS
**pI variants (n = 2)**			
pI of acidic peak	8.53–8.69	8.73	iCE
pI of main peak	8.78–8.81	8.82	
pI of basic peak	8.91–9.05	8.92–9.04	
Hydrophilic variant (n = 3)	mAb X’ is more hydrophilic than mAb X		HIC
**N-Glycan variants (n = 10)**			
Total mannosylation (%)	3.9–5.6	4.2	HPLC
Total galactosylation (%)	23.9–27.6	28.7	
Total afucosylation (%)	7.3–9.1	7.6	
**Fcγ receptor binding**^a^			
FcγRIa binding	0.73–1.17	1.12	Indirect ELISA
FcγRIIa binding	0.83–1.12	**1.45**	SPR
FcγRIIb binding	0.75–1.14	**1.31**	SPR
FcγRIIIa binding	0.73–1.32	0.90	SPR
FcγRIIIb binding	0.75–1.12	1.03	SPR
FcRn binding	0.60–1.33	0.85	SPR
C1q binding	0.75–1.11	0.95	Indirect ELISA

The range includes mAb X manufactured in-house and sourced from external agencies.

FcγRIIa and FcγRIIb Binding for mAb X’ is outside the range of mAb X.

^a^Relative binding against mAb X.

The disulfide links in mAb X’ and mAb X was assessed by non-reduced Lys-C digested peptide map LC–MS and all the eight disulfide links were found to be conserved in both the proteins. Two extra peaks were observed in the non-reduced peptide map profile of mAb X’ (Fig. S2, supplementary material), compared to the mAb X, due to the extra Lys-C digestion site in mAb X’ resulting from the E262K sequence variant. The overall secondary structure of these two antibodies was tested by far-UV CD (circular dichroism) and FT-IR (Fourier-transform infrared) spectroscopy. The far-UV CD profile of mAb X’ was similar to the profiles of mAb X lots analyzed side by side, while the contribution from different secondary structure elements determined by FTIR was also similar between mAb X’ and mAb X. Similarly, no difference was observed between the near-UV CD profiles of mAb X’ and mAb X lots, indicating similar tertiary structures in these two products. The melting temperatures obtained from the differential scanning calorimetry (DSC) studies also indicated similar unfolding patterns in mAb X and mAb X’.

**Figure 5:**
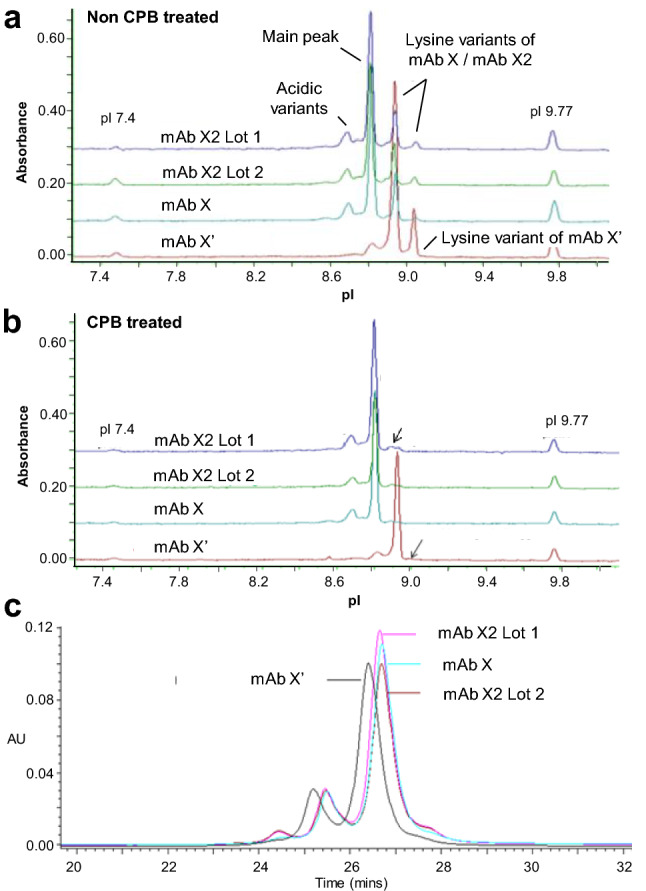
Imaged capillary electrophoresis isoelectric focusing (iCE) profiles of mAb X lots and mAb X’ (**a**) before and, (**b**) after CPB digestion. The lysine variant peaks disappeared post CPB digestion (indicated with arrows). (**c**) HIC profiles of mAb X lots and mAb X’ showing the relatively higher hydrophilicity of mAb X’. mAb X sourced from external agencies are labelled as mAb X2.

The aggregate content in mAb X’ was very low and the low molecular weight impurities, measured by non-reduced CE-SDS, was similar to the mAb X. mAb X is IgG1 and is Fc glycosylated. N-glycan profiles of both mAb X and mAb X’ was also compared and found similar. The pI of mAb X’ was more basic than mAb X due to the substitution of acidic E with basic K, and the same was evident in the pI variant analysis by imaged capillary isoelectric focusing (iCE) (Fig. 5a,b). mAb X’ showed three peaks: the first minor peak aligned with main peak of mAb X and two major peaks aligned with basic peaks B1 and B2 of mAb X; the more basic peak disappeared post carboxypeptidase B (CPB) treatment. Notably, the basicity of mAb X’ relative to mAb X in iCE analysis was not as much as seen in cation exchange chromatography. As mentioned earlier mAb X’ eluted as two peaks in cation exchange chromatography (CEX) (Fig. 4a). The second peak of the two also disappeared after CPB treatment (Fig. S3 supplementary material), indicating that the second peak is lysine variant of mAb X’.

Hydrophobic interaction chromatography (HIC) separates variants in order of increasing hydrophobicity and works orthogonal to SEC and CEX separation in principle. The HIC profile of mAb X shows four peaks where peak 3 is the main peak; peak 1 and 2 correspond to basic variants in CEX profile (data not shown). Earlier published work by John Douglass et al. also reported lysine variants as early HIC peaks^47^. Interestingly, the mAb X’ eluted slightly earlier than mAb X in HIC analysis (Fig. 5c), indicating that mAb X’ is slightly more hydrophilic than mAb X. Since E to K substitution should not enhance the hydrophilicity of the protein (actually E is slightly more hydrophilic than K), increased hydrophilicity in mAb X’ is likely to be caused by the slight structural variation of the molecule which either makes the molecule more compact making the hydrophobic residues less accessible or makes the molecule more open making hydrophilic residues more accessible. This structural variation could also lead to some differences in charge distribution of the molecule which is detected in cation exchange chromatography. However, the cIEF is run under denaturing condition and thus was not able to detect the structural variation.

The E262K substitution in mAb X’ is in the CH2 region of the antibody and thus may impact the Fc receptor binding activities of mAb X’. The fragment crystallizable γ (Fcγ) receptors and neonatal Fc receptor (FcRn) interacts with the Fc region of the mAbs and induces potent and diverse immune responses^48^. Different post-translational modifications in mAb, such as N-glycosylation, deamidation, oxidation, are known to affect the interaction with specific Fc receptors^49,50^. The relative Fc receptor binding activities of mAb X’ was assessed by Surface Plasmon Resonance (SPR) based in vitro assay, using mAb X as standard, where a relative binding potency of 0.80–1.25 is considered as similar, based on the precision of the assay. As shown in Table 1, The FcγRIa, FcγRIIIa, FcγRIIIb, FcRn and C1q binding of mAb X’ was found to be similar to mAb X. On the other hand, a marginal increase was observed in FcγRIIb binding of mAb X’, and interestingly, the FcγRIIa binding potency of mAb X’ was found to be considerably higher than mAb X. Since, E262 is not directly involved in FcγRIIa binding to the Fc^48^, the E262K substitution alone is not expected to impact the binding. Thus, this data also indicates the possibility of a structural alteration due to the E262K substitution in mAb X’, affecting the FcγRIIa binding to the mAb. This alteration seems not to be impacting global structure and thus was not captured in higher order structure assessment techniques like CD, FT-IR and DSC, but more local in nature causing change in charge distribution and surface hydrophobicity so as to be picked up by CEX and HIC techniques, respectively.

### Relative quantitation of the sequence variant by peptide mapping fingerprint and extracted ion chromatogram (PMF-EIC)

The E262K modification identified in mAb X Fc region affects the Fc receptor binding activities of the mAb in vitro and thus the same can be reflected in vivo as well, affecting the biological function. Additionally, as discussed earlier, the immunogenic effect of this substitution is unknown and very difficult to predict through any in vitro studies. Thus, control of the mAb X’ in the final drug substance and drug product is very important. To enable a downstream/purification process for removal of the mAb X’, a method is required to quantify this modification accurately at different in-process stages. The relative abundance of E262K mAb X’ can be quantified from LC–MS analysis of the trypsin digested protein, using the equation below (Eq. 1).

1 $${{\% E}}262{\text{K}} = \frac{{{\text{Area}}\,{\text{~under}}\,{\text{~E}}262{\text{K}}\,{\text{~peptide}}\,{\text{~signal~}}}}{{{\text{Area}}\,{\text{~under}}\,{\text{~E}}262{\text{K}}\,{\text{~peptide}}\,{\text{~signal~}} + {\text{~Area}}\,\,{\text{~under}}\,{\text{~parent}}\,{\text{~peptide~}}\,{\text{signal}}}}{\text{*}}100$$

where E262K peptide is V263TCVVVDVSHEDPEVK276 and Parent peptide is T260PEVTCVVVDVSHEDPEVK276

The quantification of the area under the curve from the corresponding UV signals from the tryptic peptide map profile is the simplest way; however, both the E262K peptide and parent peptide co-elutes with other peptides in the LC profile and thus quantification based on the UV signal would not be accurate enough. Complete separation of these two peptides from other peptides could not be achieved using multiple enzymes and long and shallow gradient (120 min of 2–96% of 0.09% TFA in 90:10 acetonitrile: water). Additionally, the intensity of low levels of substituted peptide was insufficient to provide good UV signal for quantitation. As a result, UV profiling could not be used for relative quantitation and signals from coupled mass spectrometer were used for this purpose. Extracted ion chromatogram (EIC) peak of the E262K and parent peptides from LC–MS were used to quantitate the area under the curves of E262K peptide and parent peptide for relative quantitation as per Eq. (1).

The PMF-EIC method was developed on LTQ Orbitrap XL mass spectrometer (ThermoFisher Scientific) to detect and quantify the E262K substitution at various in-process stages and in drug substance and in drug product to ensure effective control of E262K variant through the purification process. However, in general the PMF-EIC method has two major challenges: (1) matrix or ion suppression by co-eluting peptides; (2) ionization efficiency of the peptides due to sequence and peptide size^18,38^. Thus, the relative quantitation of E262K modification was based on the following two assumptions- (1) The ionization potential of both E262K/mutant and Native/parent peptides are similar because they are similar in size and largely share a common sequence and (2) the MS response is linear in quantitation range of both E262K peptide and parent peptide present in the sample.

The PMF-EIC method was tested for different validation parameters as per ICH guideline Q2(R1) to establish the suitability of this method for the intended purpose. Although the method was able to produce repeatable data during multiple analysis within a single day, a high relative standard deviation (~ 19%) was observed during inter-day precision study over 6 days with a sample containing ~ 0.1% E262K modified peptide (Table 2A). The specificity of this method to this particular modification was tested using the same antibody from a different source (mAb X2) and with other mAbs (mAb A and mAb B) having the same sequence in the Fc region. These mAbs do not show far basic variants in CEX analysis and thus are not expected to contain the E262K modification. Interestingly, small amounts (< 0.07%) of truncated peptide (E262K peptide) was also observed in these antibodies. Similar to the FBV of mAb X, this peptide in mAb X2, mAb A and mAb B, eluted at a different RT than the parent peptide, negating the possibility of in-source fragmentation of the parent peptide during MS analysis. This data indicates that small amount of E262K peptide (VTCVVVDVSHEDPEVK) peptide can also be generated during sample processing, as degradation product of the parent peptide (TPEVTCVVVDVSHEDPEVK). Non-specific cleavage by trypsin during 16 h digestion in Tris Cl buffer (pH 8), extended storage in auto-samplers at 4 °C and freeze − thaw of digested samples could also contribute to the degradation observed. The estimated % substitution was highly variable at these levels indicating that the detection was below the limit of quantitation (LOQ). Based on multiple inter-day analyses of the same batch of mAb X2, maximum of 0.07% substitution was observed and assigned as noise. Similar noise was also observed in other antibodies (mAb A and B) which share similar sequence in Fc region.

**Table 2:** **A** Inter-day intermediate precision of PMF-EIC method with mAb X containing ~ 0.1% of E262K substitution. The % substitution is calculated using Eq. (1). **B** Accuracy of PMF-EIC method at ~ 400 pmol and ~ 8 pmol column load.

Intermediate inter-day precision	% E262K				
**A**					
Day 1	0.08				
Day 2	0.12				
Day 3	0.14				
Day 4	0.10				
Day 5	0.12				
Day 6	0.09				
Average	0.11				
% CV	18.8				

Column load	AUC of EK peptide	AUC of native peptide	%E262K	Average %E262K	% CV
**B**					
~ 400 pmol	130,195,529	331,971,597	28.2	28.8	5.7
	128,506,560	296,820,826	30.2		
	131,613,201	339,193,814	28.0		
** ~ **8 pmol	3,463,371	2,633,558	56.8	47.5	44.3
	1,958,042	3,970,226	33.0		
	1,918,275	1,717,359	52.8		

Further, to establish the linearity and accuracy of the method, synthetic peptides were used. The E262K peptide (VTCVVVDVSHEDPEVK) and the parent peptide (TPEVTCVVVDVSHEDPEVK) were chemically synthesized and alkylated at the cysteines to mimic the E262K and parent peptides obtained during the reduced peptide map analysis. To assess the linearity of the area under the curve (AUC) obtained from E262K peptide over a dynamic range of the concentration, serial dilutions of E262K peptide was analyzed by PMF-EIC method and the signal (AUC) obtained (Fig. S4a and b, supplementary material) was plotted against the respective peptide concentration. Based on the concentration of samples injected in a typical PMF-EIC experiment (and, considering 100% cleavage by trypsin), the concentration range of the peptide was selected to mimic samples with as low as 0.01% E262K peptide. Although, a linear response was observed from the AUC of the E262K peptide over 86 fmole to 17.2 nmoles concentration range (Fig. S4c, supplementary material), the recovery (actual concentration relative to the concentration calculated from linear plot), for most of the concentration points, was far outside the generally acceptable range of 0.8–1.2 (Table S2, supplementary material). Additionally, to assess the accuracy of the method, E262K peptide and parent peptides were mixed at 1:1 molar ratio (so the expected % E262K is 50) and analyzed through the PMF-EIC method (Table 2B). At low column load (~ 8 pmol) the method was accurate enough to estimate the % E262K (47% compared to the expected 50%), however the variability among the three triplicate analysis was very high (CV = 44.3%). On the other hand, although the method was consistent (CV = 5.7%) with high column load, the estimate of % E262K was not accurate (28% compared to the expected 50%). Overall, these results indicate the limitations of this method to estimate % E262K in accurate and consistent manner and sheds reasonable doubt on the basic assumption of similar ionization potential for the two peptides and the linear response of the peptides in the quantitation range.

Based on these limitations found for PMF-EIC based method, a SRM based method was developed for accurate quantitation of E262K variant in mAb X.

### Absolute quantitation of the sequence variant by SRM based mass spectrometry (QQQ-SRM)

In an effort to develop an accurate method for more selective and sensitive detection of peptides, selective reaction monitoring (SRM) approach was utilized. In contrast to PMF-EIC, where the mass of interest is extracted post data acquisition, in SRM parent ions are exclusively selected, fragmented and dominant daughter ions can be selected to produce the final MS signal. Thus the method is very selective as the final MS signal is generated from selected daughter ions only (more commonly called as multiple reaction monitoring-MRM), while MS signals from multiple daughter ions can be added to increase sensitivity. LTQ Orbitrap, however, does not have true MRM mode and only one precursor and one of its daughter ions can be selected at a time (called as segment), thus limiting the sensitivity of this instrument in SRM mode. Nevertheless, the linearity of the SRM method was assessed in LTQ Orbitrap (Fig. S5a and b and Table S3, supplementary material) and the results were largely unsatisfactory. Although the SRM signal was reasonably linear (R^2^ = 0.98) across the EK peptide concentration range of 0.16–80 pmol, the recoveries were inconsistent and mostly outside the generally acceptable range of 0.8–1.2.

Due to unsatisfactory linearity and sensitivity in LTQ Orbitrap, the SRM method was explored on triple Quad quantitative MS instrument, TSQ Quantum Ultra (with triple Quadrupole analyzers) from Thermo. Triple-quadruple (QQQ) tandem mass spectrometer (MS/MS) provides multiple reaction monitoring (MRM) mode wherein multiple parent and daughter ions can be selected. The dominant charge states of both E262K and parent peptide were selected and subjected to fragmentation to release daughter ions. The dominant daughter ions were selected to give final signal for area quantitation against standard calibration plots (in moles) from synthetic E262K and parent peptides. The absolute quantity of E262K peptide (in moles) and the Native peptide (in moles) was then used to determine the % E262K substitution in mAb X as per the Eq. (2).

2 $${{\% ~E}}262{\text{K}} = \frac{{{\text{Moles~}}\,{\text{of}}\,{\text{~E}}262{\text{K}}\,\,{\text{~peptide~}}}}{{{\text{Moles}}\,{\text{~of}}\,{\text{~E}}262{\text{K}}\,{\text{~peptide~}} + {\text{~Moles~}}\,{\text{of~}}\,{\text{parent~}}\,{\text{peptide}}}}{\text{*}}100$$

**Figure 6:**
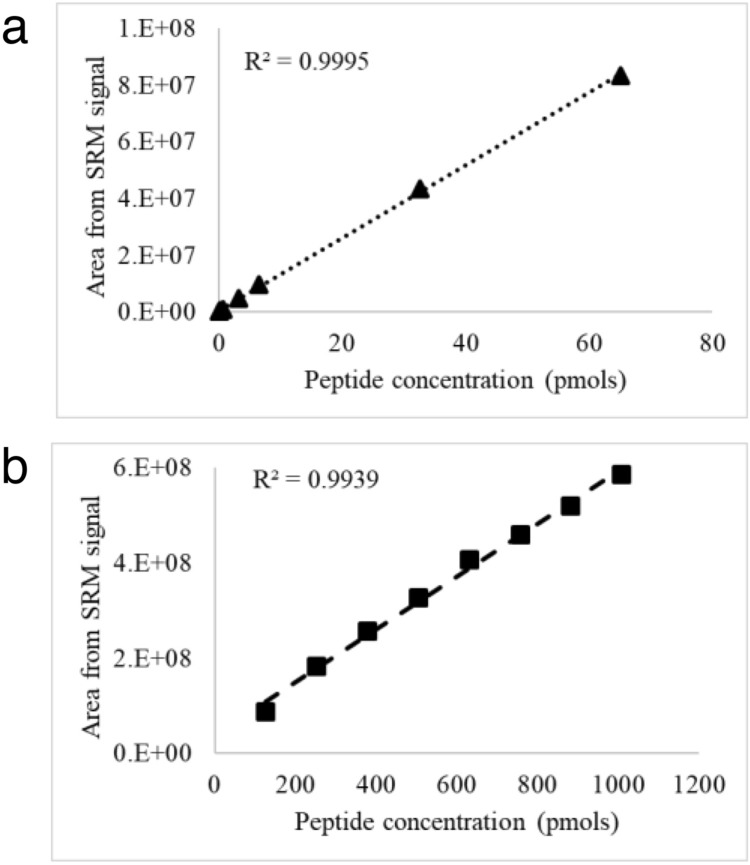
Plots showing the linearity between the peptide concentration and MS signal for (**a**) EK peptide and (**b**) native peptide by SRM based method in triple-Q MS.

The gradient method and mobile phases used in PMF-EIC was further optimized for shorter run time (30 min) and increased ionization, and the method was assessed for linearity, accuracy, precision and matrix effects using the synthetic peptides in the desired linearity range. Unlike the PMF-EIC technique, this method was not only linear over a dynamic range of peptide concentrations (Fig. 6), it was also able to measure the concentration accurately (recovery 0.9–1.1) at all the concentration points (Table 3).

**Table 3:** Recovery of EK and Native peptide in triple-Q MS using SRM.

Amount (p moles)	Peak area	Calculated amount from linear plot (pmoles)	%Recovery (actual/calculated)
**EK peptide**			
0.065	68,305	0.062	1.0
0.130	186,779	0.146	0.9
0.325	390,302	0.292	1.1
0.651	947,436	0.690	0.9
3.255	4,829,049	3.462	0.9
6.510	9,465,545	6.773	1.0
32.550	43,326,864	30.954	1.1
65.100	83,221,433	59.444	1.1
**Native peptide**			
126.187	86,146,234	118.632	1.1
252.375	182,163,787	277.256	0.9
378.562	257,317,432	401.412	0.9
504.749	327,136,398	516.756	1.0
630.936	407,050,857	648.777	1.0
757.124	459,068,585	734.712	1.0
883.311	518,580,860	833.029	1.1
1009.498	585,917,249	944.271	1.1

The precision and accuracy was evaluated at four concentration levels of quality control standards: LLOQ (lower limit of quantitation), LQC (Lower quality control), MQC (Medium quality control) and HQC (High quality control). The design of calibration curve is based on expected % E262K content in samples from antibody manufacturing process where very low levels of E262K peptide were observed in comparison to Native peptide. The acceptance criteria were adopted from regulatory guidelines for bioanalytical methods where the observed concentration should be within ± 15% of nominal value at LQC, MQC and HQC and ± 20% for LLOQ^51–53^, while four out of six (67%) of QC standards at each concentration level should pass this criterion. The results of this study is summarized in Table 4. The % CV calculated between the six analysis at LQC, MQC and HQC was within 10% for EK and native peptides, while the % CV was less than 15% at LLOQ level for both the peptides. All six analyses at LQC, MQC and HQC level with EK peptide was within the ± 15% of nominal value and four out of six analyses was within ± 20% of nominal value at LLOQ level. On the other hand, in case of the native peptide, all six analyses at LQC and MQC level and five out of the six analysis at the HQC level was within the ± 15% of nominal value and all six analyses at the LLOQ level was within ± 20% of nominal value.

**Table 4:** Accuracy and precision of EK and native peptide obtained in triple-Q MS using SRM.

Peptide	LLOQ (0.065 pmol)		LQC (0.26 pmol)		MQC (1.302 pmol)		HQC (52.08 pmol)	
	Estimated (pmoles)	Accuracy (%)	Estimated (pmoles)	Accuracy (%)	Estimated (pmoles)	Accuracy (%)	Estimate (pmoles)	Accuracy (%)
EK	0.082	26.71	0.273	5.13	1.325	1.80	46.796	− 10.15
	0.077	18.38	0.286	9.85	1.217	− 6.52	51.969	− 0.21
	0.063	− 2.57	0.287	10.22	1.320	1.41	53.371	2.48
	0.065	0.57	0.247	− 5.02	1.304	0.17	46.432	− 10.84
	0.080	23.07	0.264	1.54	1.334	2.45	48.453	− 6.96
	0.069	5.74	0.254	− 2.19	1.340	2.91	49.834	− 4.31
%CV	11.1		6.1		3.5		5.6	

Peptide	LLOQ (126.2 pmol)		LQC (378.6 pmol)		MQC (504.7 pmol)		HQC (908.5 pmol)	
Native	104.8	− 16.93	338.578	− 10.56	450.023	− 10.84	778.725	− 14.29
	137.3	8.84	345.075	− 8.85	475.883	− 5.72	739.915	− 18.56
	115.3	− 8.65	331.741	− 12.37	460.245	− 8.82	837.976	− 7.77
	111.0	− 12.00	339.177	− 10.40	460.111	− 8.84	828.069	− 8.86
	114.2	− 9.51	360.945	− 4.65	495.992	− 1.73	843.571	− 7.15
	108.9	− 13.69	338.206	− 10.66	515.043	2.04	844.758	− 7.02
%CV	9.9		2.9		5.2		5.3	

The study samples (trypsin-digested mAb X) would have multiple other tryptic peptides as background matrix to E262K and Native peptide. Moreover, shorter runtime adopted for this method (to increase through-put) resulted in co-elution of multiple peptides, which can significantly suppress the ionization of target peptides or reduce selectivity due to matrix interference. In the expected range of % E262K substitution in mAb X test samples, Native peptide is generally highly abundant and the EK peptide is present at very low amounts, the EK peptide was therefore tested for matrix interference through spike recovery. mAb X2, which has identical amino acid sequence as mAb X but does not contain the E262K, was used as E262K free matrix and six replicates at four concentration levels of QC samples (LLOQ, LQC, MQC and HQC) of EK peptide were spiked in trypsin digested mAb X2. The concentration of EK peptide on these spiked samples were estimated by SRM method and spike recovery was calculated (Table 5). At all the concentration levels at least five out of six replicates met the acceptance criteria, while the % CV between the six replicates was within the acceptable range as well. The recovery of Native peptide is discussed in Supplementary material (Table S4 and Supplementary text) However, since the % CV was on the higher side (> 10% in three out of four concentration levels), we adopted the strategy of running n = 2 independent preparations and reporting the average value only when the % CV between the two replicates is ≤ 15%, while the analysis will be repeated if the % CV between the two replicates is > 15%. This approach provided consistent results in routine analysis when the runtime of the sample sequence is not more than 20 h. Additionally, for effective removal of matrix interference and carry over in subsequent runs, the flow rate was increased to 1.5 ml/min in the wash step of the LC run which was diverted to waste.

**Table 5:** Spike recovery of EK peptide at LLOQ, LQC, MQC and HQC levels.

Peptide	LLOQ (0.065 pmol)		LQC (0.26 pmol)		MQC (1.302 pmol)		HQC (52.08 pmol)	
	Estimated (pmoles)	Accuracy (%)	Estimated (pmoles)	Accuracy (%)	Estimated (pmoles)	Accuracy (%)	Estimated (pmoles)	Accuracy (%)
EK	0.046	− 29.77	0.175	− 32.72	1.119	− 14.03	33.164	− 36.32
	0.061	− 6.40	0.246	− 5.49	1.071	− 17.76	45.91	− 11.85
	0.057	− 11.74	0.246	− 5.45	1.121	− 13.93	46.153	− 11.38
	0.063	− 3.43	0.249	− 4.19	1.161	− 10.82	47.306	− 9.17
	0.067	3.34	0.264	1.50	1.186	− 8.88	47.931	− 7.97
	0.077	19.08	0.265	2.00	ns*	−	47.027	− 9.70
%CV	16.7		13.9		3.9		12.7	

*ns, no signal observed.

Taken together, the SRM based approach in Triple-Q MS system was validated successfully for E262K SV estimation in mAb X samples with lower limit of quantitation as low as 0.007%. This was calculated considering on-column protein load (~ 65 µg) and lower limit of EK peptide calibration curve ie. 65 f. moles.

### Control of E262K substituted product during downstream purification

**Figure 7:**
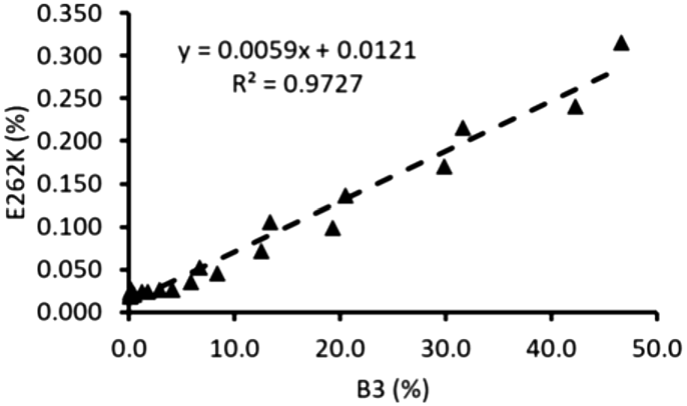
Correlation between the % B3 variant and % E262K variants in two batches of mAb X CEX fractions.

Once a sensitive method was established to quantify the E262K SV, the next step was to control the variant in the final drug product. To achieve this, protein A purified mAb X was fractionated through preparative CEX and tested on analytical CEX. As expected, the initial fractions were enriched in acidic variants and the basic variants gradually increased towards the later fractions. B1, B3 were identified as lysine variants, while B4 was characterized to be aggregates. Since B5 was characterized as E262K sequence variant, all the B5 containing fractions were discarded and rest of the fractions along with CEX load were analyzed by SRM mass spectrometry. Table 6 provides the distribution of acidic and basic charge variants and % E262K substitution in all these fractions in a representative batch of mAb X. The CEX load (inclusive of all charge variants and B5) contained 0.456% E262K substitution. Although the fractions reported here did not contain any detectable B5, trace amount of E262K substitution was still estimated in them illustrating the inconspicuous nature of the sequence variant. The E262K variant was more prominent in the later fraction and, contrary to the basic nature of this variant, early acidic fractions (F1, F2) also contained relatively higher amounts of E262K SV. The reason for this distribution could be the charge profile of sequence variant itself. Similar to mAbX, mAbX’ is also an antibody which will have its own basic and acidic species. The occurrence of E262K substitution in later basic fractions of mAbX is due to overlap of acidic variants from mAbX’. The early acidic fractions of mAbX are enriched in fragments, the E262K detected in these fractions could be fragments of mABX’ eluting there. Interestingly, excluding the early acidic fractions, a correlation between % B3 and % E262K was apparent in this analysis. The same correlation was also explored in another independent batch of mAb X and a linear relationship between % B3 and % E262K was established (Fig. 7).

**Table 6:** Charge variant and % E262K distribution in CEX fractions during mAb X purification.

Fraction number	Acidic (%)	Main (%)	B1 (%)	B2 (%)	B3 (%)	B4 (%)	B5 (%)	E262K by MS (%)
CEX load								0.456
F1	61.9	34.7	2.2	1.1	0.0	0.0	0.00	0.053
F2	42.5	53.8	2.9	0.8	0.0	0.0	0.00	0.037
F3	31.5	63.0	4.5	1.0	0.0	0.0	0.00	0.030
F4	24.7	68.6	5.9	0.8	0.0	0.0	0.00	0.018
F5	20.2	71.7	7.4	0.7	0.1	0.0	0.00	0.022
F6	16.9	73.4	9.4	0.1	0.1	0.0	0.00	0.016
F7	14.2	74.4	11.2	0.1	0.1	0.0	0.00	0.020
F8	7.9	70.0	19.7	1.2	1.3	0.0	0.00	0.012
F9	2.6	44.6	38.1	3.8	10.3	0.6	0.00	0.055
F10	1.8	44.1	42.3	7.4	22.8	1.5	0.00	0.122
F11	1.7	15.5	37.0	9.6	34.7	1.4	0.00	0.223
CEX pool (F3 to F8)								0.012
Drug substance								0.014

Far basic variant was annotated as basic 5 variant.

Generally, sequence variant at < 0.1% level at a single site is considered to be acceptable to make sure that the sequence variants in total remain below a threshold (1–2%)^4,7^. However, a very conservative approach was taken here and only the fractions containing ≤ 0.050% E262K substitution was considered for pooling, which corresponds to ≤ 10% B3, as per the linear correlation established between B3 and % E262K. Having B3 below 10% also helped in controlling basic charge variants in the final drug product. Thus, in addition to the established pooling criteria to control the product quality attributes such as fragment, aggregate, deamidation etc., this criteria (B3 ≤ 10%) was also applied to pool the CEX fractions for further processing. Thus fraction F3 to F8 were pooled for the batch illustrated in Table 6 and the final drug product obtained had % E262K substitution as low as 0.014.

This approach was used to control the E262K variant in ten consecutive batches of mAb X and the SV was controlled to under 0.04% in all these batches. Further, pre-clinical toxicology study conducted in monkeys with multiple doses of mAb X having ~ 0.080% E262K did not reveal any product specific safety findings. Taken together, the highly sensitive SRM method enabled the control of E262K variant to a level where it does not impart any efficacy and safety concern.

## Discussion

In this communication, we have reported the identification and characterization of a sequence variant in monoclonal antibody based therapeutic and developed two different LC–MS/MS based approaches to estimate the SV. The more sensitive technique between these two, the SRM based approach in a QQQ mass spectrometer, was validated and further utilized to control the SV in the final drug product during downstream purification process.

While next generation sequencing (NGS) and software based SV searches in high resolution LC–MS/MS data generated from the enzymatic peptide map analysis of drug product or upstream products are used widely to identify sequence variants in therapeutic proteins, both of these techniques have challenges, especially when the SV is present in trace amounts. NGS can be time and cost consuming and may result in false positives, while trace amounts of SV may evade the software based search due to lack of sufficient MS or MS/MS data. However, an approach combining NGS and LC–MS/MS, where all the hits resulted from NGS analysis can be further verified by targeted processing of LC–MS/MS data, can be the most reliable approach for SV identification in a product or clone. In absence of NGS capabilities, LC–MS/MS based characterization of enriched charge variants was utilized here to identify any sequence variants in mAb X. The E to K substitution at 262 position in heavy chain Fc region was identified by tryptic peptide map LC–MS/MS analysis of the far basic variant (FBV) and this finding was further validated by Glu-C digested peptide map LC–MS analysis. The E262K containing mAb X (mAb X’) was purified (~ 98%) from CEX and characterized by an array of physicochemical and Fc related functional assays. Interestingly, although E is more hydrophilic than K, the mAb X’ was appeared to be more hydrophilic than mAb X in HIC analysis, indicating the possibility of a structural difference between these two variants. This observation was further substantiated by the differences observed in FcγRIIa binding capabilities of these two products. Since E262 is not known to be directly involved in FcγRIIa interaction, it is more likely that a structural alteration due to E262K modification in mAb X’ is affecting the FcγRIIa binding potency. Additionally, the apparent structural alterations may also lead to the far basic nature of mAb X’. As evident from the iCE analysis of mAb X and mAb X’, the pI of mAb X’ is similar to the one lysine variant of mAb X (Fig. 6a). However, in CEX analysis the mAb X’ elutes much later than the one lysine variant (B1) of mAb X (Fig. 5a). The separation in CEX depends on the accessible charge of the protein and the accessible charge may depend on the structure of the protein. Certain structural changes may expose relatively charged residues to the column resulting in a change in the column-protein interaction and thus these variants may elute differently. Hence, the relatively strong basic nature of mAb X’ may signify certain structural modification in the SV containing protein. However, this structural alteration was not detected in Far and Near UV CD, FT-IR and DSC indicating that the global structure may not be impacted. At this moment the exact location of this suspected structural modification is not clear and high resolution methodologies such as hydrogen–deuterium exchange mass spectrometry (HDX-MS) can be used further to pin-point the exact region of the apparent structural alteration.

Since the modified (SV containing) mAb X elutes as far basic variant in analytical CEX, the same separation technique can be used during the downstream purification to control the SV in drug substance and drug product. To enable this approach highly sensitive Mass spectrometry based methods were developed to estimate the trace amount of SV. Although the peptide map LC–MS-extracted ion chromatography (PMF-LC–MS-EIC) based method is a relatively simple and widely used for MS based PTM/variant analysis, this method was not able to estimate the E262K variant with acceptable accuracy and consistency. This method presumes that the peptides involved in % variant calculation (native and EK peptide, in this case) ionize similarly under the given mass spectrometry conditions. However, the method validation results indicate that this assumption may not be true and the three amino acid difference between these two peptides may bring in some differences in mass spec ionization potential, leading to inconsistent and inaccurate data. The alternate approach, SRM based method in a Triple-Q MS, was found to be much more sensitive and accurate. This method depends on the absolute quantification (in pmoles) of the native and EK peptides based on the parent and daughter ions specific to these two peptides. Additionally, the SRM based method was designed to be a shorter one and thus providing a better turn-around-time (TAT) for in-process sample analysis during the downstream purification. This method was successfully validated and used as in-process control to limit the E262K content in the purified mAb X. All the CEX fractions generated during the CEX purification step was analyzed by SRM based method and only the fractions containing insignificant amounts (≤ 0.05%) of SV was pooled to proceed further. Generally, the CEX fractions are pooled based on certain product quality attributes such as aggregate, fragments, charge variants etc. and results in some loss of the product. The additional pooling criteria (% E262K ≤ 0.07 and % B3 ≤ 10, based on the correlation between % E262K and % B3) imposed here ensured insignificant amounts (< 0.04%) of E262K SV in the drug product and it was utilized to generate mAb X consistently in the lab and at the pilot and manufacturing scales. Animal toxicity studies was conducted in Cynomolgus monkeys with a drug product with ~ 0.08% E262K SV and no toxic reactions were reported. Further, the same approach was endorsed by regulatory agencies for manufacturing drug products for clinical use. Interestingly, although the SRM based method was able to detect and quantify the SV in all the CEX fractions, the SV was below detection level of the PMF-EIC method in many fractions. The PMF-EIC method was not able to detect the SV in the drug product as well. This observation further emphasizes the importance of developing a very sensitive technique to estimate trace amounts of sequence variants.

Overall, sequence variants are considered to be undesired for the bio-therapeutics and appropriate measures should be taken to control SVs at the very early stage of the product development. While a combination of NGS and HRMS can be a tool for early detection of SVs at the clone level, the time and cost associated with a reliable NGS assessment may make this approach non-accessible for all the developmental programs, especially at the early stage. In those scenario, thorough characterization of enriched product variants through multiple analytical techniques can provide reliable information on the nature of different variants present in the product, including the sequence variants. Further, as described here, the inherent chemical and structural nature of the SV can be utilized to purify out the variant containing product and availability of a very sensitive analytical technique to reliably estimate trace amounts of SV is pivotal to this approach. To our knowledge, such an extensive characterization of sequence variant in antibody biopharmaceutical and its control in the final drug product using mass spectrometry has not been demonstrated earlier. At times the clone producing the highest titer and a product with desirable quality attributes may contain trace amounts of SV and rejecting the clone right away may impart serious business implications. Thus, the approach presented here can be utilized to understand the properties of the SV extensively and based on the assessment, sensitive techniques and strategies can be designed to control the SV in the purified drug product.

## Methods

### Samples and materials

The IgG1 mAbs X, X’, A and B were expressed in standard CHO cells and purified using standard antibody purification procedures at Biocon. No animals were used for experimentation. mAb X2 was sourced from external agency. The list of reagents and other materials used is described in Supplementary material. Reagents and materials used in analytical techniques were procured from various vendors as described below. Dithiothreitol (DTT), Tris base [tris(hydroxymethyl)aminomethane)], trifluoroacetic acid (TFA), acetic acid (glacial), calcium chloride dihydrate, and hydrochloric acid used in sample processing were purchased from Sigma-Aldrich and Guanidine hydrochloride and iodoacetamide (IAM) were obtained from Sigma. Trypsin (sequencing-grade) was purchased from Promega and LysC (sequencing grade modified) was obtained from Roche. Acetonitrile from J.T. Baker was used in mobile phases. Deionized water (18 MΩ cm at 25 °C) for mobile phases was prepared using a Millipore’s Milli-Q purification system. Customized peptides: VTCVVVDVSHEDPEVK (EK peptide) and TPEVTCVVVDVSHEDPEVK (Native peptide) were custom synthesized from GenScript (Piscataway, NJ). C-13 and N-15 labelled Valine containing EK peptide: V*TCVVVDVSHEDPEVK and Native peptide TPEV*TCVVVDVSHEDPEVK were used as internal standards and custom synthesized from Polypeptide (France). * indicates C-13 and N-15 labelling of Valine. Primers atgatctcccggacccctgaggtcacatgcgtggtggtggacgtg and atgatctcccggacccctaaggtcacatgcgtggtggtggacgtg were obtained from Life Technologies.

### Intact mass analysis

Intact antibody samples were diluted to a concentration of 1 mg/mL with 0.1% TFA in 50: 50 acetonitrile: water and analyzed using reverse-phase LC–MS on Waters ACQUITY UPLC with a photo diode array (PDA) detector coupled to Waters Synapt high definition mass spectrometry (HDMS) system equipped with an ESI source. The samples were injected on an ACE5 C4 column (100 × 2.1 mm) for chromatographic separations. Mobile phase A was 0.1% Formic acid in Milli-Q water and mobile phase B was acetonitrile. Elution was achieved using a 10 min gradient of 10–90% of acetonitrile. Flow rate and column oven temperature were set at 200 μL/min and 40 °C, respectively, throughout the run. Mass spectrometric analysis was carried out in positive ion mode. Scan range of 2000–4000 m/z was used along with 3.00 kV capillary voltage and 40 V as cone voltage. Desolvation gas temperature was set to 300 °C and source temperature was 120 °C. Trap and transfer collision energy values were 5 V each. Instrument was calibrated in the m/z range of 150–4000 using Sodium Iodide. Deconvolution of the ESI mass spectra was done using Max Ent 1 algorithm in Mass Lynx v4.1 software. The mass range used for deconvolution was 145,000–155,000, minimum intensity ratio left and right being 20%. Damage model was “Uniform Gaussian” and width at half height was 2.4. Number of iterations was set to 15.

### Reduced mass analysis

Intact antibody samples were denatured with Guanidium hydrochloride (final concentration of 3 M), reduced with DTT (final concentration of 10 mM) at 37 °C for 1 h and diluted to a final concentration of 1 mg/mL with 0.1% TFA in 50% acetonitrile. The samples were injected on an ACE 5 C4-300 (100 × 2.1 mm; 5 μm particle size; 300 Å pore size) column for chromatographic separations. Mobile phase A was 0.1% Formic acid in Milli-Q water. Elution was achieved using a 27 min gradient of 10- 50% acetonitrile as Mobile phase B. Flow rate was set at 150 μL/min for elution step and 200 μL/mL for washing step. Column temperature was maintained at 40 °C throughout the run. Mass spectrometric analysis was carried out in positive ion mode. Scan range of 500–4000 m/z was used along with 3.00 kV capillary voltage and 25 V as cone voltage. Desolvation gas temperature was set to 300 °C and source temperature was 120 °C. Trap and transfer collision energy values were 5 V each. Instrument was calibrated in the m/z range of 150–4000 using Sodium Iodide. Deconvolution of the ESI mass spectra was done using Max Ent 1 algorithm in Mass Lynx v4.1 software. The mass range used for deconvolution was 20,000–60,000, minimum intensity ratio left and right being 20%. Damage model was “Uniform Gaussian” and width at half height was 1.2. Number of iterations was set to 15.

### Peptide mass fingerprinting—EIC method

Intact antibody samples were denatured using guanidium chloride (final concentration of 3 M), reduced using DTT (final concentration of 10 mM) at 37 °C for 1 h and then alkylated using IAM (final concentration of 20 mM) at 37 °C for 1 h. After alkylation, the samples were desalted using a size exclusion GE HiTrap Desalting (5 mL) column at a flow rate of 0.3 mL/min using 0.05% TFA in 40:60 acetonitrile: water as the mobile phase. The protein eluting from the column was collected in a microcentrifuge tube and concentrated in a Savant SPD121P SpeedVac concentrator (Thermo Scientific). The optical density (OD) of the samples was determined by recording the absorbance at 280 nm and correcting for any light scattering at 340 nm using a spectrophotometer and the final concentration of the protein (mg/mL) was calculated from the OD reading using extinction co-efficient of 1.64 (theoretical extinction coefficient based on the confirmed amino acid sequence). The desalted sample equivalent to 250 µg of collected protein was concentrated further for digestion with trypsin up to a final volume of 70 μL. Trypsin (1:25 w/w) was added to the sample after adjusting the sample pH to 8.0 and the sample was incubated at 37 °C for 16 h to obtain the peptide mixture. This peptide mixture was separated using RP LC–MS on a Shimadzu UFLC coupled to LTQ Orbitrap XL (ThermoFisher Scientific) mass spectrometer. Mobile phase A was 0.1% TFA in Milli-Q water and mobile phase B was 0.09% TFA in 90:10 acetonitrile: water. 125 μg of the peptide mixture was injected on an ACE 5 C18-300 (250 × 4.6 mm; 5 μm particle size; 300 Å pore size) column, separated at 40 °C using a 120 min gradient of 2- 96% Mobile phase B at a flow rate of 0.8 mL/min. The eluting peptides were detected using a UV detector at 215 nm followed by mass spectrometry using LTQ Orbitrap XL in positive mode. The MS system was calibrated in the m/z range of 100–2000 using Thermo Scientific Pierce LTQ ESI Positive Ion Calibration Solution (mixture of caffeine, MRFA and Ultramark 1621 in a solution of acetonitrile, methanol, and acetic acid). MS/MS analyses were performed in a data-dependent mode with one cycle of scans consisting of one full MS scan of m/z range 100- 2000 in profile mode using the FTMS analyzer (resolution = 30,000), followed by MS/MS of the fragment ions using the ion trap analyzer in profile mode at a normal scan rate. Ion selection for MS/MS was done using an isolation width of 1 Da, then fragmentation was done by collision induced dissociation (CID) with helium gas using normalized collision energy of 35, activation Q of 0.25 and activation time of 30 ms. The default charge state was set at 2. Quantification of the area corresponding to the EIC signals of EK peptide and Native/parent peptides was done using QualBrowser within Xcalibur v 2.5.5 SP1 (ThermoFisher Scientific). Mass range for extraction of native/parent peptide was 1070–1073 m/z and 906.45–908.45 m/z for the EK peptide covering full isotopic distribution for both peptides. Integration of the peptide peaks was done using the ICIS algorithm.

### Peptide mass fingerprinting—SRM method

Intact antibody samples were denatured using guanidium chloride (final concentration of 3 M), reduced using DTT (final concentration of 10 mM) at 37 °C for 30 min and then alkylated using IAM (final concentration of 20 mM) at 37 °C for 1 h. Desalting, OD estimation, sample concentration, and trypsin digestion was done as described above. After adding trypsin, the samples were incubated at 37 °C for 5 h. At the end of 5 h, it was diluted with cold diluent (2% acetic acid in 20: 80 acetonitrile: water, kept at 2- 8 °C) and injected on an ACE 5 C18- 300 (250 × 4.6 mm; 5 μm particle size; 300 Å pore size) column maintained at 40 °C. Mobile phase A consisted of 1% acetic acid in Milli-Q water and Mobile phase B was acetonitrile. Flow rate of 0.8 mL/min was used during the peptide elution step and 1.5 mL/min was used for the washing step and the divert valve was used to divert the flow to waste during the higher flow rate. A 13 min gradient of 6- 45% acetonitrile (3% per min) was used for elution of the peptides. The peptides were detected TSQ Quantum ultra AM mass spectrometer (ThermoFisher Scientific) equipped with an ESI source. The MRM transitions used were 245.75 Da (± 0.2 m/z) for parent ion 604.67 Da from EK peptide and 226.53 Da, 327.61 Da, 471.70 Da (± 0.2 m/z) for parent ion 1070.01 Da from Native peptide. Instrument parameters were optimized separately for EK and Native peptides and therefore both peptides were detected in separate segments. Data was acquired in positive mode with Centroid data type. Scan width (0.2 m/z), scan time (0.02 s), peak width (Q1 and Q3: 0.70 FWHM), number of micro scans (1) and collision gas pressure (1.5 mTorr) were kept common for both EK and Native/parent peptides. Collision energy of 22 V was used for EK peptide and 50 V was used for Native/parent peptide. Spray voltage of 3500 V, vaporizer temperature of 300 °C, sheath gas and auxiliary gas pressures of 60 mTorr and 20 mTorr, respectively, and capillary temperature of 275 °C were other instrument parameters optimized to get maximum peptide signal. LC Quan v 2.5 was used for data processing. ICIS peak detection algorithm was used for optimal peak integration.

### SNP detection using cast-PCR (competitive allele-specific Taqman qPCR) technique

SNP leading to E262K substitution was detected using cast-PCR (Competitive allele-specific Taqman qPCR) technique as described previously^46^. Briefly, the cDNA extracted from mAb X clone was analyzed by qPCR using primers specific to wild type (atgatctcccggacccctgaggtcacatgcgtggtggtggacgtg) and SNP containing genomic DNA (atgatctcccggacccctaaggtcacatgcgtggtggtggacgtg). Standard TaqMan™ thermocycling conditions were used: 10 min at 95 °C, 40 cycles of 20 secs at 95 °C, 45 secs at 60 °C. Amplification (ΔRn vs cycle) was determined from standard amplification plot.

### Preparation of calibration curve using peptide standards

Synthetic peptides for EK and Native/parent sequences were used as standards for the quantification of % EK in unknown samples. The working stock of the native/parent and EK peptides was prepared separately by denaturing (using guanidium hydrochloride), reducing (using DTT) and alkylating (using IAM) 1 mg/mL master stock solution and further diluting to 0.12 mg/mL (Native) and 0.04 mg/mL (EK) using the diluent (2% acetic acid in 20:80 acetonitrile: water). Master stock solution was prepared by dissolving the lyophilized powder of respective synthetic peptides (Native and EK) in 50 mM Tris HCl buffer with 1 mM Calcium Chloride (pH 8.0) to get 1 mg/mL solution.

Tables S5 and S6 show the scheme of preparation of standards for calibration curve and quality control standards of EK and Native/parent peptides from respective working stock solutions.

For recovery experiments 100 µL of appropriate standard was added to 900 µL of mAb A’ trypsin digested sample and 50 µL was injected on HPLC. For Native peptide, to reduce contribution of inherent Native peptide in mAb A’ matrix, 500 µL of appropriate standard was added to 500 µL of mAb A’ trypsin digested sample and 50 µL was injected on HPLC.

*Internal standards spiking:* 5000 ppb levels of EK and Native internal standards were spiked into each calibration standard and samples.

### Non reduced peptide mapping using Lys C

Disulphide mapping analysis was performed on Waters ACQUITY UPLC coupled to Waters Synapt HDMS system. 100 µg of intact antibody was denatured using 6 M guanidine hydrochloride at 37 ºC for 30 min. 1 ml of the cooled Ethanol is added and stored in − 20 °C for 1 h for precipitation of the protein. The sample is centrifuged at 8000 rpm for 15 min and collected precipitate was treated with 50 µl of 2 M Urea, 2 mM CaCl_2_, 0.2 M Tris HCl (pH 6.5) and 2.5 µg of Lys C enzyme (Roche sequencing grade modified; reconstituted with MilliQ water) in the ratio of 1: 20 (Lys C: antibody, w/w). The reaction mixture was incubated at 37 °C for 48 h. The digested sample was further analyzed LC MS. Standard operating conditions were used for LC MS as described below:

- Mobile phase A: 100% acetonitrile.
- Mobile phase B: 0.1% FA in water.
- Column: C18, 2.1 × 100 mm, 1.7 µm, part no: 1860002352.
- Flow rate: 0.3 ml/min.
- Column temp: 40 °C.

#### LC Gradient program

**Table:** 

Time (min)	% B
0	99
75	70
83	15
86	15
87	99
90	99

#### Mass spectrometric parameters

- Analyzer mode: sensitivity.Analyzer mode: sensitivity.
- Cone voltage: 25 V.
- Scan time: 1 s.
- Mode: positive.
- Mass range: 50–2500 m/z.
- Trap collision: 4–30 V.

### Size exclusion chromatography

40 µg of antibody was separated on TSK gel G3000W XL 7.8 mm ID* 300 mm, 5 µ column using mobile phase 20 mM sodium phosphate, 0.25 M NaCl, pH 7.4 at isocratic flow rate of 0.5 ml/min for 35 min at 25 °C.

### Cation exchange chromatography

80 µg of antibody was separated on Dionex ProPac WCX-10 4 mm ID* 250 mm, 5.0 µm using gradient described below at 1 ml/min at 25 °C.

- Mobile phase A: 10 mM phosphate buffer pH 7.5
- Mobile phase B: 10 mM phosphate buffer 100 mM NaCl pH 7.6

**Table:** 

Time (min)	% A	%B
0	100	0
6.5	100	0
54.5	32	68
54.6	0	100
60	0	100
60.1	100	0
70	100	0

### Hydrophilic interaction chromatography

5 µg of antibody (1 mg/ml) were separated on Tosoh TSK butyl NPR 4.6X10cm (2.5 µ) at 25 °C using below gradient scheme at 0.5 ml/min and 220 nm.

Mobile phase A: 100 mM Sodium phosphate (30 mM sodium dihydrogen phosphate, 70 mM disodium hydrogen phosphate), 1.3 M ammonium sulphate pH 6.5

Mobile phase B: 100 mM sodium phosphate (70 mM sodium dihydrogen phosphate, 30 mM disodium hydrogen phosphate) pH 6.4.

**Table:** 

Time (min)	%B
0	0
6.0	00
50.0	54
50.1	100
58.0	100
58.1	0
75.0	0

### Far UV and near UV CD

The antibody samples were analyzed in CD spectrophotometer Jasco J- 815 in both Far UV (measure range 200–260 nm) and near UV (measure range 260–360 nm) region for secondary structure and tertiary structure analysis, respectively. 0.4 mg/ml sample was analyzed in 1 mm path length quartz cuvette with CD acquisition parameters of 0.1 nm data pitch, standard sensitivity, 1 nm bandwidth, 1 s DIT, 200 nm/min scanning speed and 6 accumulations. Same parameters were used for acquiring near UV CD with 4 mg/ml of sample in cuvette with 10 mm path length.

### ATR FT-IR

The Attenuated Total Reflectance-Fourier Transform-Infrared spectrum of 25 mg/ml antibody samples was acquired in Jasco FT/IR 6300 type A in scan range of 7800–350 cm^−1^ with 8 cm^−1^ resolution and 256 accumulations. Rest of the parameters were on auto mode.

### Differential scanning calorimetry

The intact antibody was diluted to 2 mg/ml using placebo and was loaded on to the sample holder whereas reference holder is loaded with the respective placebo. The spectrum was acquired for temperature scan range of 20–100° C at 30 °C per hour scan rate.

### Intrinsic fluorescence spectroscopy

0.2 mg/ml of antibody for intrinsic fluorescence with excitation at 278 nm and emission spectrum recorded from 300 to 400 nm at scan rate of 600 nm/min. Average of 10 scans was stored as final spectrum. Both excitation and emission slit width was kept at 5 mm.

### Non reduced capillary electrophoresis using sodium dodecyl sulphate (NR CESDS)

CE analysis was performed on Sciex PA 800 Plus instrument using 30 cm capillary with separation voltage of 18 kV applied for 30 min. The antibody was desalted using 10 kDa MCWO nanosep at 8000 rpm and diluted to 1 mg/ml using SDS sample buffer. 2 µl of 10 kDa internal standard, 5 µl of 250 mM iodoacetamide was added to the reaction volume of 100 µl. and incubated at 70 °C for 3 min. The reaction mixture was spun at 8000 rpm for 8 min to remove air bubbles and transferred to CE universal vials. The samples were electrokinetically injected at 10 kV for 25 secs. 32 karat software was used for processing electropherogram.

### Imaged capillary iso-electric focusing

iCE analysis was performed on ProteinSimple iCE 280 using focusing period of 2 min at 1500 V followed by 5 min at 3000 V. The 10 mg/ml of antibody was desalted using 10 kDa MCWO nanosep using MilliQ at 13,000 rpm. To 5 µl of desalted antibody 185 µl of 0.35% methyl cellulose gel (Protein Simple) containing 8 M urea, 7 µl of pharmalyte 3–10, 3 µl of pharmalyte 8–10.5 (GE heathcare), 0.2 µl of pI marker 9.77 and 7.40 (Protein Simple) was added. The mixture was vortexed and spun at 8000 rpm for 8 min to remove air bubbles and transferred to CE universal vials.

### Fc binding using SPR based capture format

Affinity to recombinant human FcγRIIa, FcγRIIb and FcγRIIIa were determined using surface plasmon resonance (SPR) with a Biacore T200/T100 (GE Healthcare). A penta- His antibody (Qaigen) was covalently immobilized on a CM5 chip using standard amine coupling chemistry for a specified contact time. A constant concentration of recombinant FcγRs (FcγRIIa, FcγRIIb or FcγRIIIa) was captured on the anti-His surface as the ligand and different concentrations of analyte mAb X or mAb X’ were passed at a constant flow rate for set association and dissociation times. As the analyte binds to the ligand, Fc receptor immobilized on the surface, accumulation of protein on the surface results in an increase in refractive index. This change in refractive index is measured in real time, and the result plotted as response or resonance units (RUs) versus log concentration of mAb X or mAb X’. Relative Binding was determined using parallel line analysis in Stegmann Systems software.

### Fc binding using SPR based direct format

Affinity to recombinant human FcγRIIIb and neonatal Fc receptor (FcRn) were also determined using surface plasmon resonance (SPR) with a Biacore T200/T100. The Fc receptor was covalently immobilized on a CM5 chip using standard amine coupling chemistry for a specified contact time. The analyte mAb X or mAb X’ was injected in aqueous solution through the active and reference flow cells, under continuous flow. As the analyte binds to the ligand, Fc receptor immobilized on the surface, accumulation of protein on the surface results in an increase in refractive index. This change in refractive index is measured in real time, and the result plotted as response or resonance units (RUs) versus log concentration of mAb X or mAb X’. Relative Binding was determined using parallel line analysis in Stegmann Systems software.

### ELISA based FcγRIa binding

Affinity to recombinant human FcγRIa was determined using an ELISA based format where FcγRIa was coated on the ELISA plate followed by serially diluted mAb X or mAb X’. This binding was detected using a HRP tagged goat anti-human F(ab′)2 specific antibody which converts the chromogenic substrate, 3,3′,5,5′-tetramethylbenzidine (TMB) to form a blue colored product. The reaction was stopped by addition of dilute sulphuric acid (1 N). The final colored product was read at 450 nm/ 630 nm and the corrected absorbance (A450–A630 nm) value was plotted versus log concentration of mAb X or mAb X’. Relative Binding was determined using parallel line analysis in Stegmann Systems software.

### ELISA based C1q binding

An ELISA format was utilized for determining C1q binding. 96 well plates were first coated with serially diluted mAb X or mAb X’, followed by a defined constant amount of C1q protein and biotinylated anti-C1q antibody was added. The presence of captured biotinylated anti-C1q antibody was detected using horse-radish-peroxidase (HRP) conjugated streptavidin which converted the chromogenic substrate, 3,3′,5,5′-tetramethylbenzidine (TMB), forming a blue colored product. The reaction was stopped and Relative Binding calculated as described in FcγRIa binding.

## Supplementary Information

Supplementary Information.

## References

[1] Li J, Zhu Z (2010). Research and development of next generation of antibody-based therapeutics. Acta Pharmacol. Sin..

[2] Deng N, Zhou H, Fan H, Yuan Y (2017). Single nucleotide polymorphisms and cancer susceptibility. Oncotarget.

[3] Yang Y (2010). Detecting low level sequence variants in recombinant monoclonal antibodies. MAbs.

[4] Douglass JV (2019). Biopharmaceutical industry practices for sequence variant analyses of recombinant protein therapeutics. PDA J. Pharm. Sci. Technol..

[5] U.S. Food and Drug Administration. Guidance for Industry Q6B Specifications: Test Procedures and Acceptance Criteria for Biotechnological/Biological Products. U.S. Department of Health and Human Services Food and Drug Administration. Center for Drug Evaluation and Research (CDER), Center for Biologics Evaluation and Research (CBER), 1999. https://www.fda.gov/media/71510/download.4.

[6] Lian Z, Wu Q, Wang T (2016). Identification and characterization of a-1 reading frameshift in the heavy chain constant region of an IgG1 recombinant monoclonal antibody produced in CHO cells. MAbs.

[7] Harris C (2019). Identification and characterization of an IgG sequence variant with an 11 kDa heavy chain C-terminal extension using a combination of mass spectrometry and high throughput sequencing analysis. MAbs.

[8] Fu J (2012). Characterization and identification of alanine to serine sequence variants in an IgG4 monoclonal antibody produced in mammalian cell lines. J. Chromatogr. B..

[9] BLA 761028 ABP215, a proposed biosimilar to US-Avastin. ODAC Briefing Document. July 13, 2017.

[10] Feeney L (2013). Eliminating tyrosine sequence variants in CHO cell lines producing recombinant monoclonal antibodies. Biotechnol. Bioeng..

[11] Yu XC (2009). Identification of codon-specific serine to asparagine mistranslation in recombinant monoclonal antibodies by high-resolution mass spectrometry. Anal. Chem..

[12] Zeck A (2012). Low level sequence variant analysis of recombinant proteins: an optimized approach. PLoS ONE.

[13] Li Y (2016). Characterization of alanine to valine sequence variants in the Fc region of nivolumab biosimilar produced in Chinese hamster ovary cells. MAbs.

[14] Griaud F (2017). Identification of multiple serine to asparagine sequence variation sites in an intended copy product of LUCENTIS® by mass spectrometry. MAbs.

[15] Zhang Z, Shah B, Bondarenko PV (2013). G/U and certain wobble position mismatches as possible main causes of amino acid misincorporations. Biochemistry.

[16] Que AH (2010). Sequence variant analysis using peptide mapping by LC–MS/MS. BioProcess Int..

[17] Borisov, O. V., Alvarez, M.,Carroll, J. A., & Brown, P. W. *Sequence Variants and Sequence Variant Analysis in Biotherapeutic Proteins. State-of-the-art and emerging technologies for therapeutic monoclonal antibody characterization. Bio-pharmaceutical characterization: the NISTmAb case study.* ACS Symposium series 1201; American Chemical Society. Vol 2, pp. 63–117 (2015).

[18] Wong HE, Huang CJ, Zhang Z (2018). Amino acid misincorporation in recombinant proteins. Biotechnol. Adv..

[19] Zhang S, Bartkowiak L, Nabiswa B, Mishra P, Fann J (2015). Identifying low-level sequence variants via next generation sequencing to aid stable CHO cell line screening. Biotechnol. Prog..

[20] Wright C (2016). Genetic mutation analysis at early stages of cell line development using next generation sequencing. Biotechnol. Prog..

[21] Lin TJ (2018). Evolution of a comprehensive, orthogonal approach to sequence variant analysis for biotherapeutics. MAbs.

[22] Huang, Y. et al. Identification and quantification of signal peptide variants in an IgG1 monoclonal antibody produced in mammalian cell lines *J. Chromatogr. B. Anal. Technol. Biomed. Life Sci.* **1068–1069**, 193–200 (2017). 10.1016/j.jchromb.2017.08.046 29078145

[23] Neill A (2015). Characterization of recombinant monoclonal antibody charge variants using offgel fractionation, weak anion exchange chromatography, and mass spectrometry. Anal. Chem..

[24] Liu H, Ren W, Zong L, Zhang J, Wang Y (2019). Characterization of recombinant monoclonal antibody charge variants using WCX chromatography, icIEF and LC-MS/MS. Anal. Biochem..

[25] Ponniah G (2015). Characterisation of the acidic species of a monoclonal antibody using weak cation exchange chromatography and LC-MS. Anal. Chem..

[26] Mouchahoir T, Schiel JE (2018). Development of an LC-MS/MS peptide mapping protocol for the NISTmAb. Anal. Bioanal. Chem..

[27] Andrews GL, Dean RA, Hawkridge AM, Muddiman DC (2011). Improving proteome coverage on a LTQ-orbitrap using design of experiments. J. Am. Soc. Mass Spectrom..

[28] Harris RP, Mattocks J, Green PS, Moffatt F, Kilby PM (2012). Determination and control of low level amino acid misincorporation in human thioredoxin protein produced in a recombinant *Escherichia coli* production system. Biotechnol. Bioeng..

[29] Ren D (2011). Detection and identification of a serine to arginine sequence variant in a therapeutic monoclonal antibody. J. Chromatogr. B..

[30] Brady LJ, Scott RA, Balland A (2015). An optimized approach to the rapid assessment and detection of sequence variants in recombinant protein products. Anal. Bioanal. Chem..

[31] Traylor MJ (2016). Comprehensive discovery and quantitation of protein heterogeneity via lc-ms/ms peptide mapping for clone selection of a therapeutic protein. Anal. Chem..

[32] Kil, Y. J. et al. *Towards a Comprehensive Bioinformatic Analysis of the NIST Reference mAb. State-of-the-Art and Emerging Technologies for Therapeutic Monoclonal Antibody Characterization Volume 3. Defining the next generation of analytical and biophysical techniques.* ACS Publications, pp. 395–414 (2015).

[33] Genedata Expressionist (https://www.genedata.com/products/expressionist/).

[34] Eng JK, McCormack AL, Yates JR (1994). An approach to correlate tandem mass spectral data of peptides with amino acid sequences in a protein database. J. Am. Soc. Mass Spectrom..

[35] Fenyo D, Beavis RC (2003). A method for assessing the statistical significance of mass spectrometry-based protein identifications using general scoring schemes. Anal. Chem..

[36] Bern, M., Kil, Y. J., & Becker, C. Byonic: advanced peptide and protein identification software. *Curr. Protoc. Bioinform.* Chapter13 Unit 13.20 (2012). 10.1002/0471250953.bi1320s40 PMC3545648 23255153

[37] Zhang Z (2009). Large scale identification and quantification of covalent modifications in therapeutic proteins. Anal. Chem..

[38] Annesley TM (2003). Ion suppression in mass spectrometry. Clin. Chem..

[39] Loos G, Van Schepdael A, Cabooter D (2016). Quantitative mass spectrometry methods for pharmaceutical analysis. Philos. Trans. Ser. Proc. Math. Phys. Eng. Sci..

[40] Li W, Wypych J, Duff RJ (2017). Improved sequence variant analysis strategy by automated false positive removal. MAbs.

[41] Zhang Y, Wen ZH, Washburn MP, Florens L (2009). Effect of dynamic exclusion duration on spectral count based quantitative proteomics. Anal. Chem..

[42] Dick LW, Qiu D, Mahon D, Adamo M, Cheng KC (2008). C-terminal lysine variants in fully human monoclonal antibodies: investigation of test methods and possible causes. Biotechnol. Bioeng..

[43] Bremer ETVD (2015). Human IgG is produced in a pro-form that requires clipping of C-terminal lysines for maximal complement activation. MAbs.

[44] Xie H (2010). Rapid comparison of a candidate biosimilar to an innovator monoclonal antibody with advanced liquid chromatography and mass spectrometry technologies. MAbs.

[45] Swaney DL, Wenger CD, Coon JJ (2010). The value of using multiple proteases for large-scale mass spectrometry-based proteomics. J. proteome Res..

[46] Morlan J, Baker J, Sinicropi D (2009). Mutation detection by real-time PCR: a simple, robust and highly selective method. PLoS ONE.

[47] Douglass JV, Wallace A, Balland A (2008). Separation of populations of antibody variants by fine tuning of hydrophobic-interaction chromatography operating conditions. J. Chromatogr. A..

[48] Paul A (2011). Ramsland, structural basis for FcγRIIa recognition of human IgG and formation of inflammatory signaling complexes. J. Immunol..

[49] Houde D, Peng Y, Berkowitz SA, Engen JR (2010). Post-translational modifications differentially affect IgG1 conformation and receptor binding. Mol. Cell Proteomics..

[50] Geuijen KPM (2017). Rapid screening of IgG quality attributes—effects on Fc receptor binding. FEBS Open Bio.

[51] European Medicines Agency. Guideline on bioanalytical method validation. EMEA/CHMP/EWP/192217/2009 Rev. 1.

[52] Bioanalytical Method Validation: Guidance for Industry FDA 2018.

[53] Tiwari G, Tiwari R (2010). Bioanalytical method validation: an updated review. Pharm Methods..

