# Author Correction: Identification, characterization and control of a sequence variant in monoclonal antibody drug product: a case study

**DOI:** 10.1038/s41598-021-96916-1

**Published:** 2021-08-30

**Authors:** Anushikha Thakur, Rekha Nagpal, Avik Kumar Ghosh, Deepak Gadamshetty, Sirisha Nagapattinam, Malini Subbarao, Shreshtha Rakshit, Sneha Padiyar, Suma Sreenivas, Nagaraja Govindappa, Harish V. Pai, Ramakrishnan Melarkode Subbaraman

**Affiliations:** 1 Research and Development (RND), Biocon Biologics Limited, Biocon Park, SEZ, Plot No. 2&3, Phase-IV, Bommasandra Industrial Estate, Bommasandra‑Jigani Link Road, Bangalore, 560099 India; 2 Divis Laboratories Limited, Chotuppal, Hyderabad 508252 India; 3 Sunquest Information Systems, Bangalore, 560095 India; 4 Alvotech Hf, Saemundargata 15‑19, 101 Reykjavik, Iceland

Correction to: *Scientific Reports* 10.1038/s41598-021-92338-1, published online 24 June 2021

The Supplementary Information published with this Article contained errors.

The order of authors was incorrectly given as Anushikha Thakur, Rekha Nagpal, Deepak Gadamshetty, Sirisha N., Malini Subbarao, Shreshtha Rakshit, Sneha Padiyar, Suma Sreenivas, Nagaraja G., Harish V Pai, Ramakrishnan M. S, Avik Kumar Ghosh.

In addition, Affiliation 1 was incorrectly given as “Science and Technology Innovation Center (SnTIC), Biocon Biologics, Biocon Park, SEZ, Bommasandra Industrial Area, Jigani Link Road, Bangalore, India 560100.”

These errors have now been corrected in the Supplementary Information file that accompanies the original Article.

