# Supplementary material for: Identification, characterization and control of a sequence variant in monoclonal antibody drug product: a case study
Source: Sci Rep. 2021 Jun 24;11:13233. doi: 10.1038/s41598-021-92338-1 (PMC8225904; doi:10.1038/s41598-021-92338-1)
Supplement: Supplementary file 1 — Supplementary Information. [file 41598_2021_92338_MOESM1_ESM.docx]

**Identification, characterization and control of a sequence variant in monoclonal antibody drug product: A Case Study**

Anushikha Thakur^1,5^, Rekha Nagpal^1,5^, Avik Kumar Ghosh^4^ , Deepak Gadamshetty^2^, Sirisha Nagapattinam^3^, Malini Subbarao^1^, Shreshtha Rakshit^1^, Sneha Padiyar^1^, Suma Sreenivas^1^, Nagaraja Govindappa^1^, Harish V. Pai^1^, Ramakrishnan Melarkode Subbaraman^1,6^

^1^ Research and Development (RND)

Biocon Biologics Limited, Biocon Park

SEZ, Plot No. 2&3, Phase-IV

Bommasandra Industrial Estate

Bommasandra- Jigani Link Road

Bangalore, 560099, India

^2^Divis laboratories limited

Chotuppal, Hyderabad, India 508252

^3^Sunquest Information systems

Bangalore, India 560095

^4^Alvotech hf.

Saemundargata 15-19

101 Reykjavik

Iceland

^5^Both authors contributed equally

^6^Correspondence should be addressed to: [ramakrishnan.melarkode@biocon.com](mailto:ramakrishnan.melarkode@biocon.com) (ORCID: [0000-0003-1977-3014](https://orcid.org/0000-0003-1977-3014))

**Running title:** Identification and characterization of a sequence variant in mAb

**Supplementary information**

**Recovery of Native peptide in QQQ method**

The blank matrix was not possible for Native peptide as it is a constant region conserved sequence and the MabX2 sample used for the analysis contained pre-existing Native peptide at levels closer to LQC level of the qualified calibration curve. The spiking on top of it at HQC levels led to signal saturation and thus, recoveries for Native peptide were calculated at LQC and MQC level by subtracting the pre-existing peak area from observed peak area. The difference observed between the estimated concentration and the actual concentration of native peptide are described in Table S4.

**Supplementary Tables**

**Table S1.** Intact and Reduced mass analysis of isolated main variant (MV) and far basic charge variant (FBV) of MabX.

| **mAb X charge variant** | **Observed Mass*** |
| --- | --- |
| **Intact Mass** | |
| Far basic charge variant | 148081 (G0F/G0F)  148337 (G0F/G0F+2K) |
| Main variant | 148082 (G0F/G0F) |
| **Light Chain Mass** | |
| Far basic charge variant | 23413 |
| Main variant | 23412 |
| **Heavy Chain Mass** | |
| Far basic charge variant | 50646 (G0F)  50775 (G0F+K) |
| Main variant | 50646 |

***** Method variability: ± 50 ppm

**Table S2.** Recovery of E262K peptide in PMF-EIC method.

| **Amount of E262K peptide (pmole)** | **Area under the curve** | **Concentration of E262K peptide (pmole) calculated from linear plot** | **Recovery (Actual concentration/concentration calculated from plot)** |
| --- | --- | --- | --- |
| 0.086 | 84356 | Not Reported | Not Reported |
| 0.86 | 217907 | Not Reported | Not Reported |
| 8.6 | 1626398 | Not Reported | Not Reported |
| 86 | 16092462 | Not Reported | Not Reported |
| 860 | 141779150 | 1360 | 1.6 |
| 8600 | 874403365 | 9545 | 1.1 |
| 17200 | 1519321386 | 16749 | 1.0 |

**Table S3.** Recovery of E262K peptide in SRM method in Orbitrap LTQ.

| **Concentration of E262K peptide (pmole)** | **Area under the curve** | **Concentration of E262K peptide (pmole) calculated from linear plot** | **Recovery (Actual concentration/concentration calculated from plot)** |
| --- | --- | --- | --- |
| 0.16 | 0 | Not Reported | Not Reported |
| 1.12 | 4118 | 1 | 0.7 |
| 1.6 | 5690 | 3 | 1.9 |
| 8 | 8789 | 7 | 0.9 |
| 16 | 19345 | 22 | 1.4 |
| 80 | 60905 | 79 | 1.0 |

**Table S4.** Recovery of Native peptide at LQC and MQC of the calibration curve. ‘ns’ refers to no signal. Recovery is also calculated as % Difference.

| **Dilution integrity** | **Obtained Native p moles** | **Pre-existing (in matrix) Native p moles** | **Calculated Native p moles** | **% Difference** |
| --- | --- | --- | --- | --- |
| MQC (504.749 p moles) | 779.178 | 245.586 | 533.592 | 5.71 % |
|  | 704.192 |  | 458.606 | -9.14 % |
|  | 670.242 |  | 424.656 | -15.87 %$ |
|  | 668.662 |  | 423.076 | -16.18 %$ |
|  | 655.83 |  | 410.244 | -18.72 %* |
|  | 693.348 |  | 447.762 | -11.29 % |
| LQC (378.562 p moles) | 590.715 |  | 345.129 | -8.83 % |
|  | 563.158 |  | 317.572 | -16.11 %$ |
|  | 522.027 |  | 276.441 | -26.98 %* |
|  | 568.041 |  | 322.455 | -14.82 % |
|  | 594.622 |  | 349.036 | -7.80 % |
|  | ns |  | - | - |

*$ Marginally outside; * Outside acceptance criteria*

**Table S5. a** Scheme of dilution for preparation of standards for calibration curve of EK peptide. Injection volume: 50µL. **b** Scheme of dilution for preparation of QC standards for accuracy and precision of EK peptide. Injection volume: 50µL.

**a**

| **Calibration standard ID** | **Volume of EK working stock solution (µL)** | **Volume of Diluent (µL)** | **Amount of EK peptide on column (p moles)** |
| --- | --- | --- | --- |
| EK 8 | 59 (of EK working stock) | 941 | 65.100 |
| EK 7 | 100 (of EK 8) | 100 | 32.550 |
| EK 6 | 100 (of EK 8) | 900 | 6.510 |
| EK 5 | 100 (of EK 6) | 100 | 3.255 |
| EK 4 | 100 (of EK 6) | 900 | 0.651 |
| EK 3 | 100 (of EK 4) | 100 | 0.325 |
| EK 2 | 100 (of EK 4) | 400 | 0.130 |
| EK 1 | 100 (of EK 4) | 900 | 0.065 |

**b**

| **Calibration standard ID** | **Volume of EK working stock solution (µL)** | **Volume of Diluent (µL)** | **Amount of EK peptide on column (p moles)** |
| --- | --- | --- | --- |
| LLOQ | 100 (EK 4) | 900 | 0.065 |
| LQC | 400 (EK4) | 600 | 0.260 |
| MQC | 200 (EK 6) | 800 | 1.302 |
| HQC | 800 (EK 8) | 200 | 52.08 |

**Table S6. a** Scheme of dilution for preparation of standards for calibration curve of Native peptide. Injection volume: 50µL. **b** Scheme of dilution for preparation of QC standards for accuracy and precision of Native peptide.

**a**

| **Calibration standard ID** | **Volume of Native working stock solution (µL)** | **Volume of Diluent (µL)** | **Amount of Native peptide on column (p moles)** |
| --- | --- | --- | --- |
| Native 8 | 270 (Native ws) | 480 | 1009.498 |
| Native 7 | 175 (Native 8) | 25 | 883.311 |
| Native 6 | 75 (Native 8) | 25 | 757.124 |
| Native5 | 25 (Native 8) | 75 | 630.936 |
| Native 4 | 150 (Native 8) | 150 | 504.749 |
| Native 3 | 75 (Native 4) | 25 | 378.562 |
| Native 2 | 50 (Native 4) | 50 | 252.375 |
| Native 1 | 25 (Native 4) | 75 | 126.187 |

**b**

| **Calibration standard ID** | **Volume of Native working stock solution (µL)** | **Volume of Diluent (µL)** | **Amount of Native peptide on column (p moles)** |
| --- | --- | --- | --- |
| LLOQ | 250 (Native-4) | 750 | 126.187 |
| LQC | 750 (Native-4) | 250 | 378.562 |
| MQC | 500 (Native-8) | 500 | 504.749 |
| HQC | 900 (Native-8) | 100 | 908.548 |

**Supplementary Figures**

**Figure S1. a** Schematic of generation of GluC peptide L_237_LGGPSVFLFPPKPKDTLMISRTPK/R_262_ and VTCVVVDVSHEDPE_276_ from substituted peptide L_237_LGGPSVFLFPPKPKDTLMISRTPK/R_262_VTCVVVDVSHEDPE_276_ in mAb X.

***
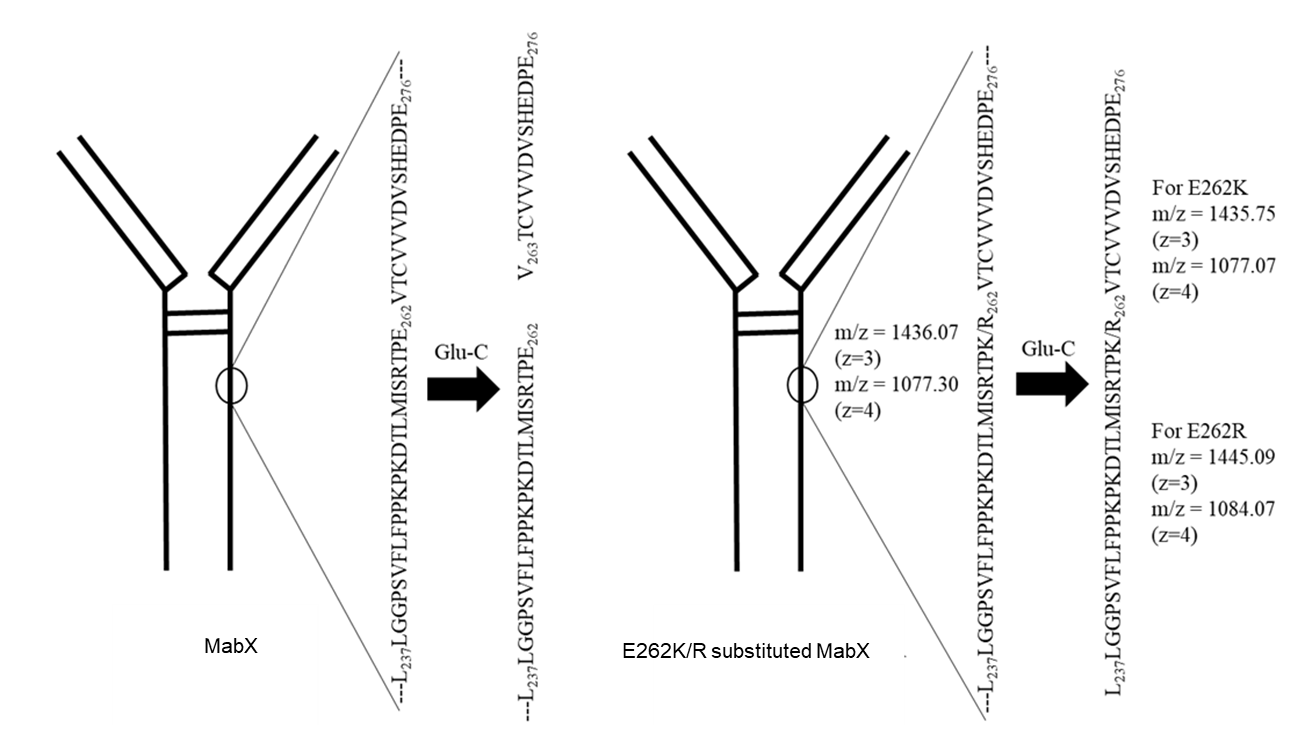
***

**Figure S1. b** Extracted ion chromatogram of E262K substituted GluC peptide L_237_LGGPSVFLFPPKPKDTLMISRTPK/R_262_VTCVVVDVSHEDPE_276_ in FBV showing Z=3 and Z=4 charge states.

**
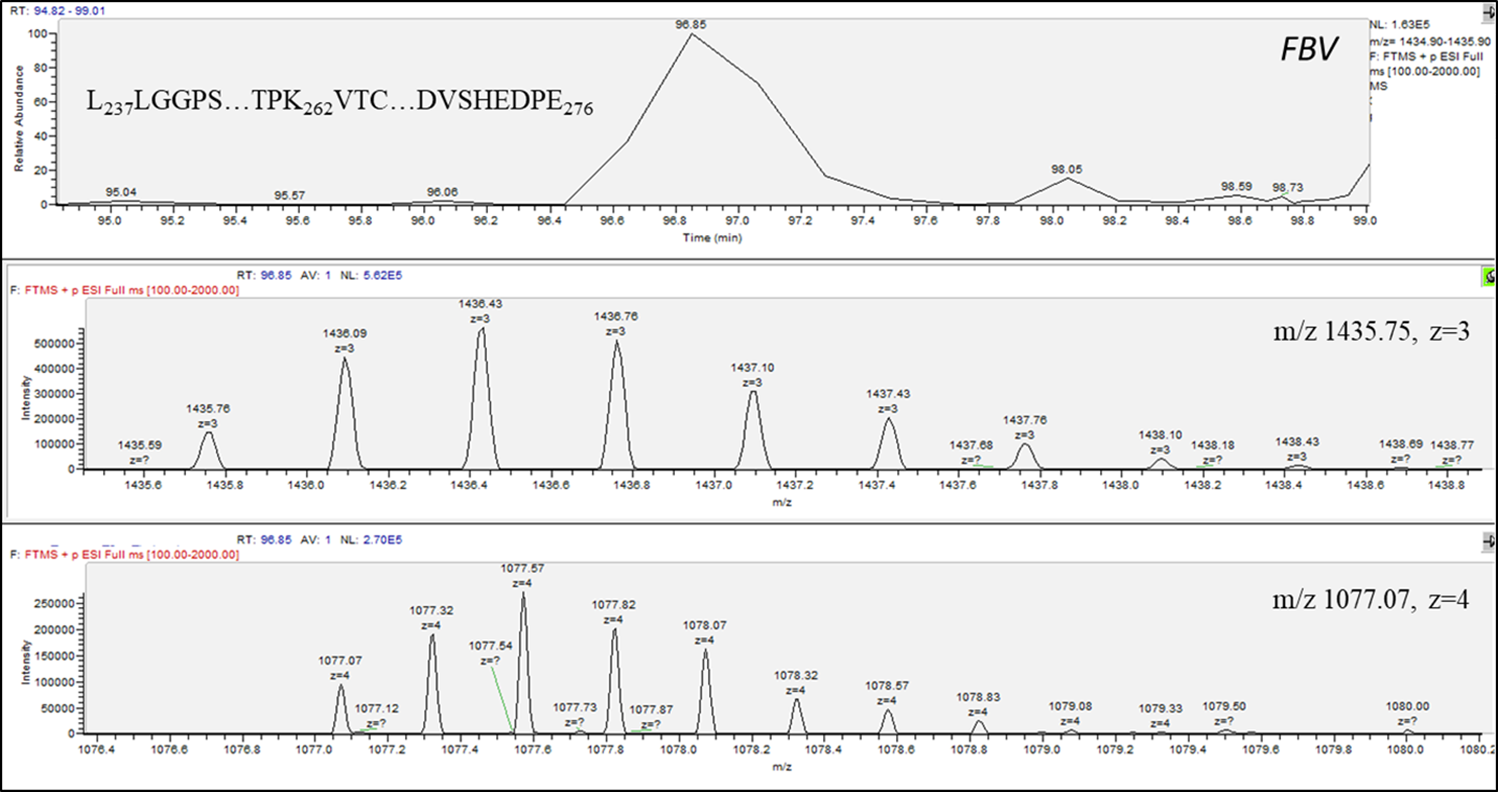
**

**
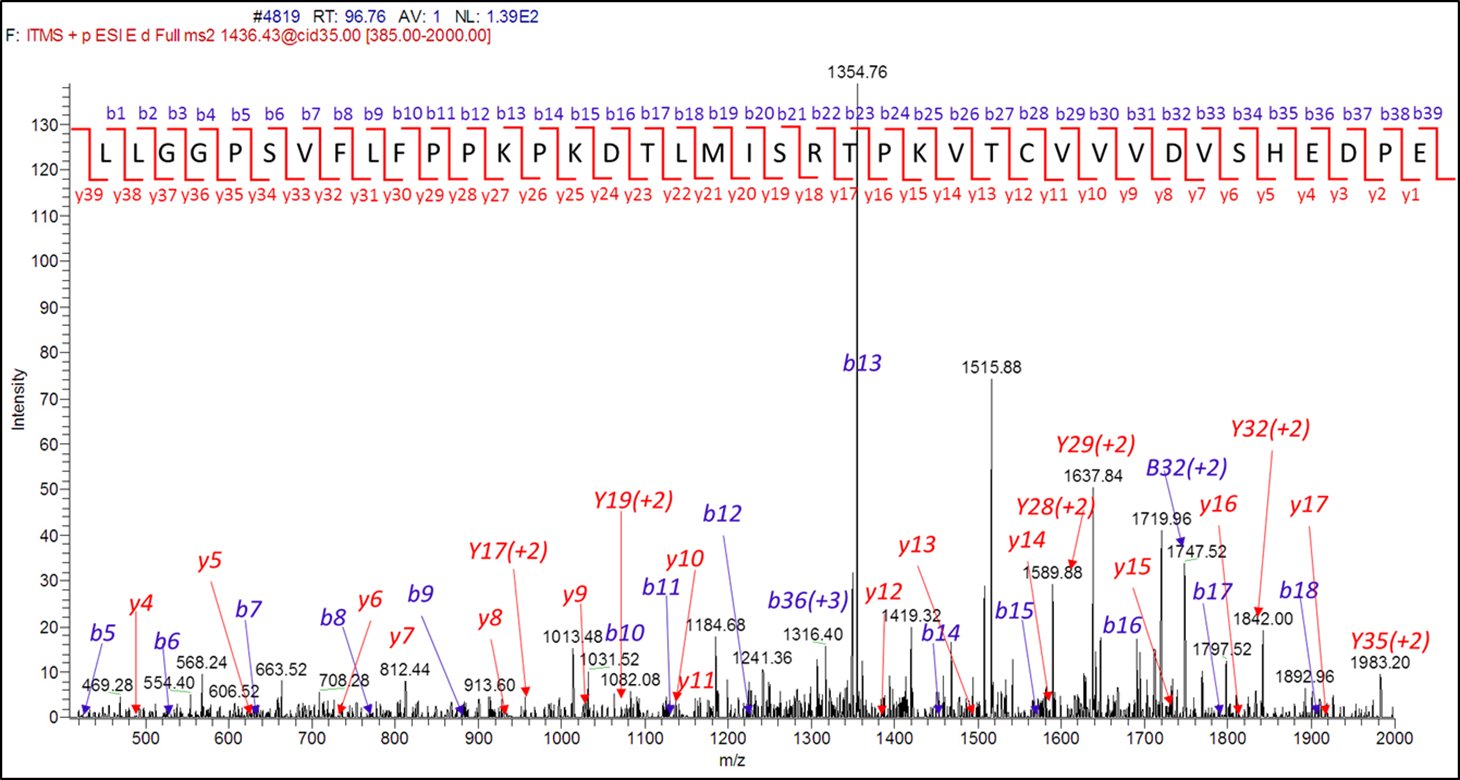
Figure S1. c** MS/MS analysis of the GluC digested peptide containing E262K substitution at m/z 1436.43 (z=3) and retention time 96.76 mins elucidating the amino acid sequence.

**Figure S2. a** Schematic showing LysC digested peptide E1 and disulphide linked peptide E2 generated as a result of E262K substitution in mAb X’ but absent in mAb X. **b** Non-reduced LysC peptide map profiles (BPI) of mAb X and MabX’, showing the two extra peaks (E1 and E2) in MabX’ profile.

**a**


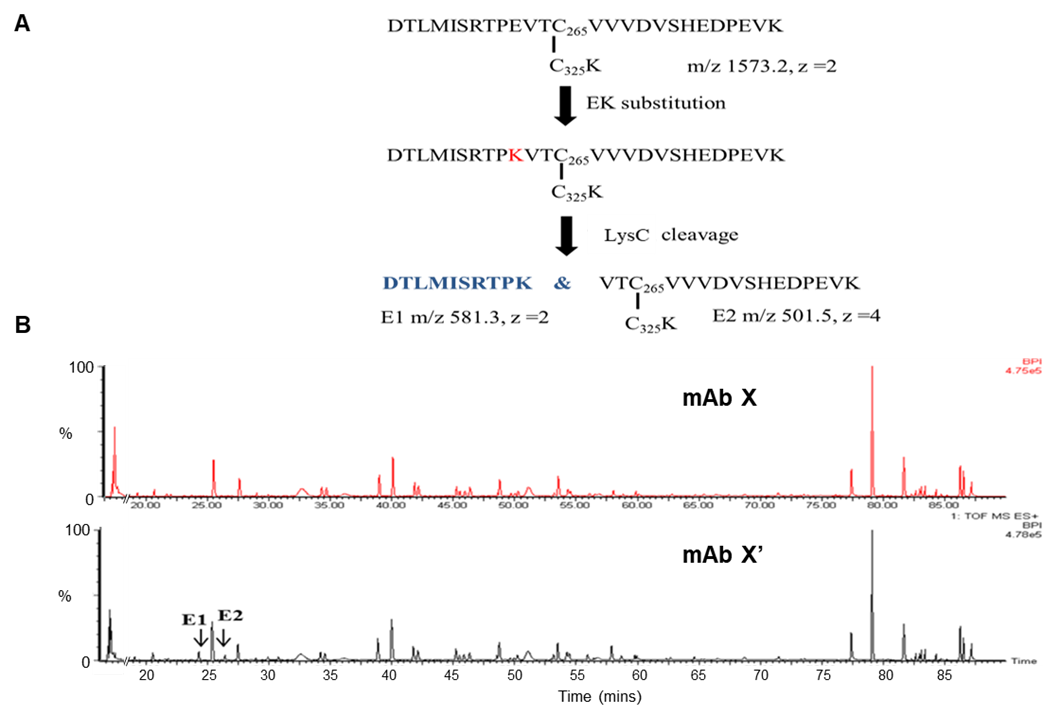


**b**

**
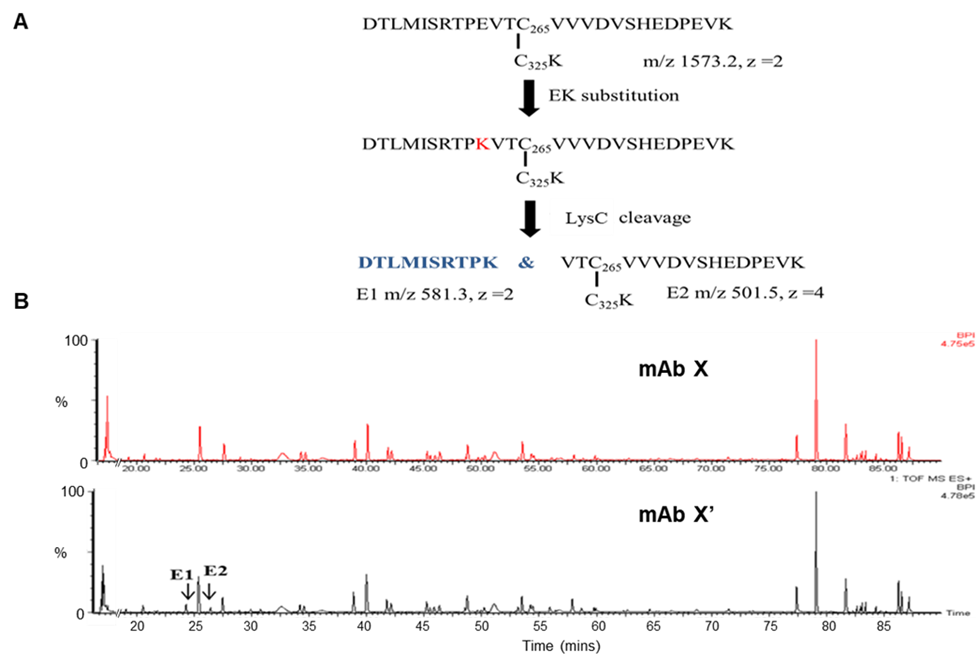
**


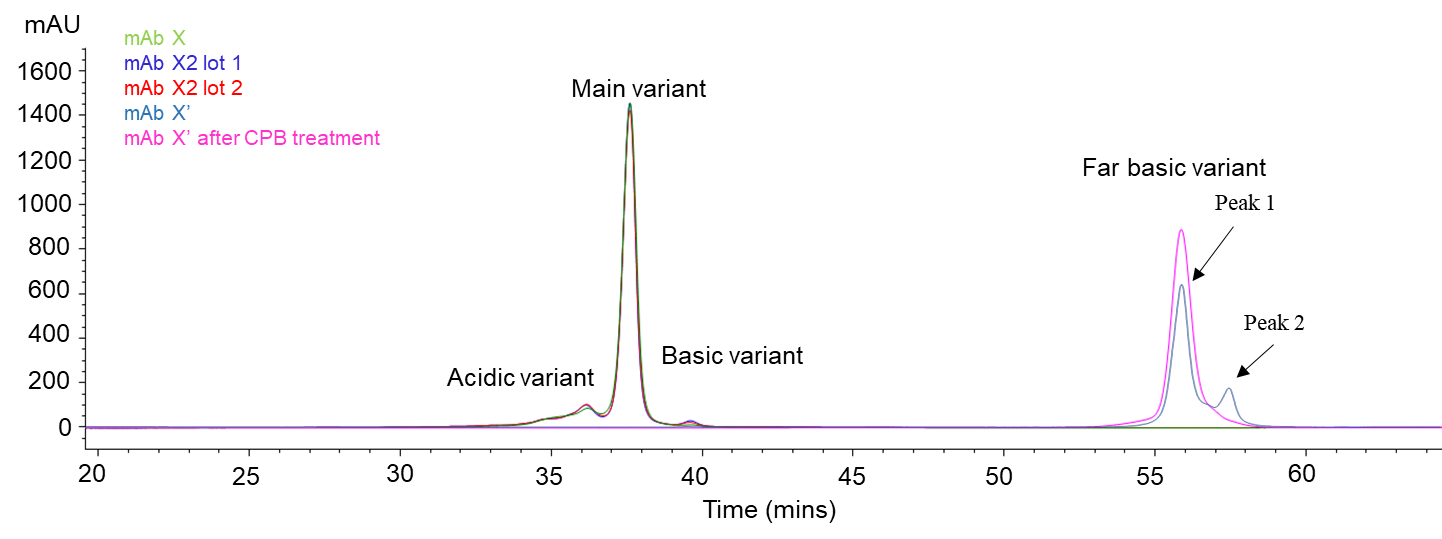
**Figure S3.** Overlay of charge variant profile of mAb X’ before and after CPB digestion along with mAb X2. mAbX’ is enriched far basic variant observed as Peak 1 and 2 where peak 2 is lysine variant of peak 1 as confirmed by CPB treatment.

**Figure S4.** Detection of EK peptide using PMF-EIC mode in Orbi-Trap mass spectrometer. **a** EIC and, **b** MS of EK peptide serially diluted from 17.2 nmoles to 86 fmoles. **c** Plot showing the linearity of the EIC area of EK peptide against the amount of the peptide

**
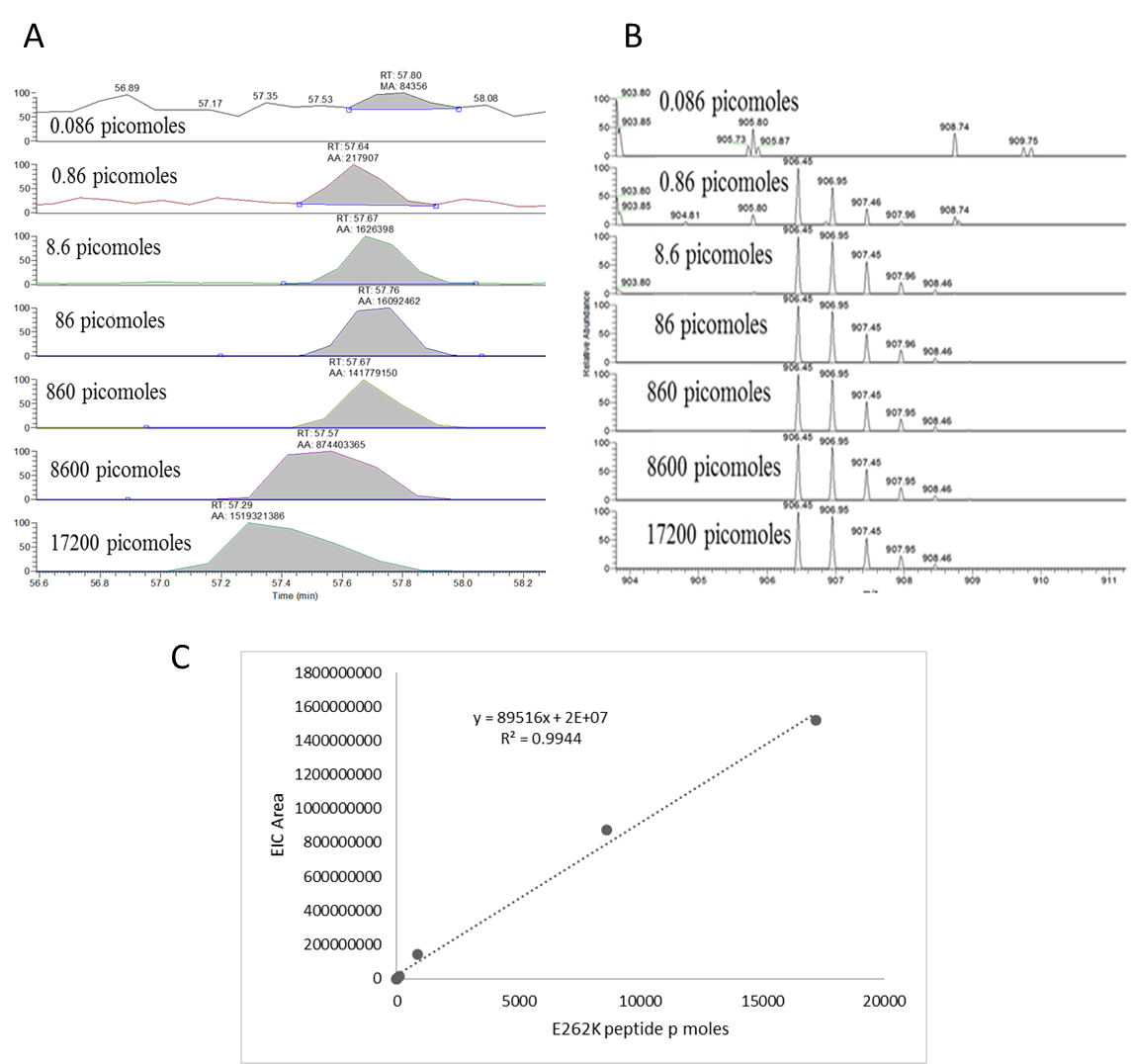
a b**

**c**

**
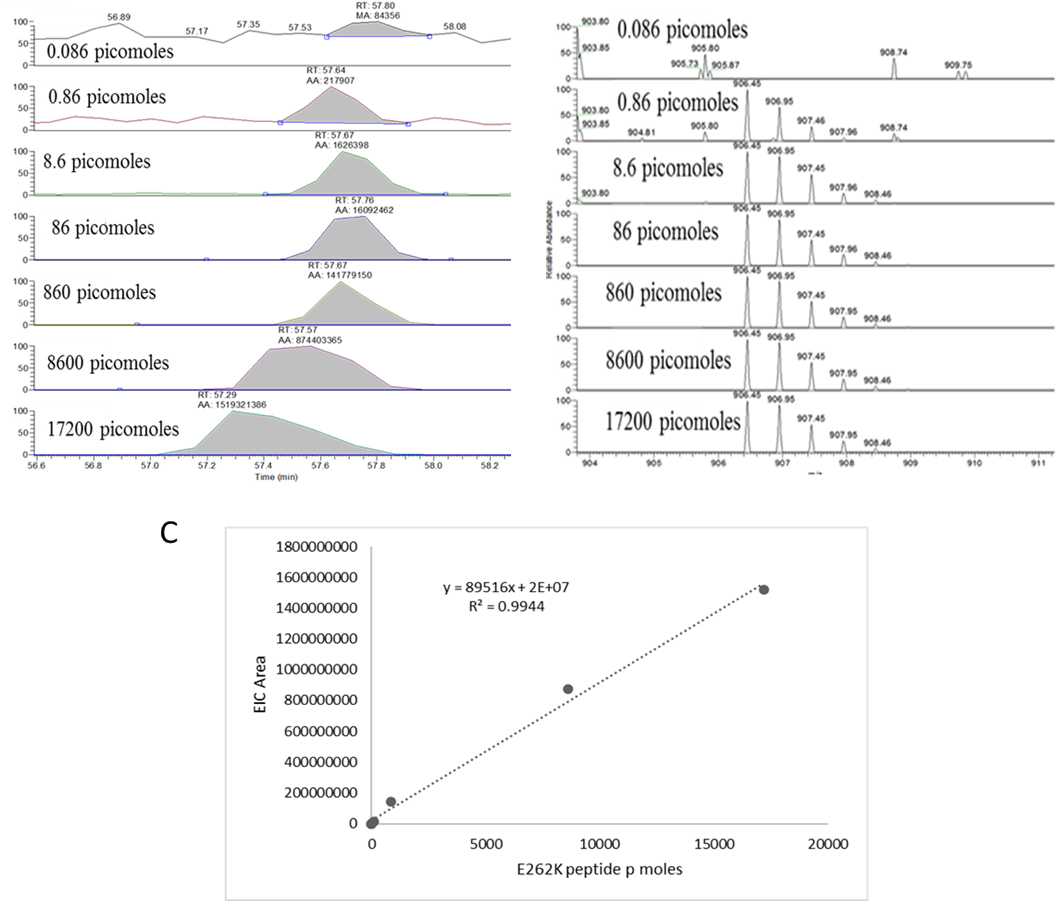
**

**Figure S5.** Detection of EK peptide using SRM mode in Orbi-Trap mass spectrometer. **a** SRM signal of EK peptide serially diluted from 80 to 0.16 pmoles. **b** Plot showing the linearity of the SRM signal from the EK peptide against the amount of the peptide.

**
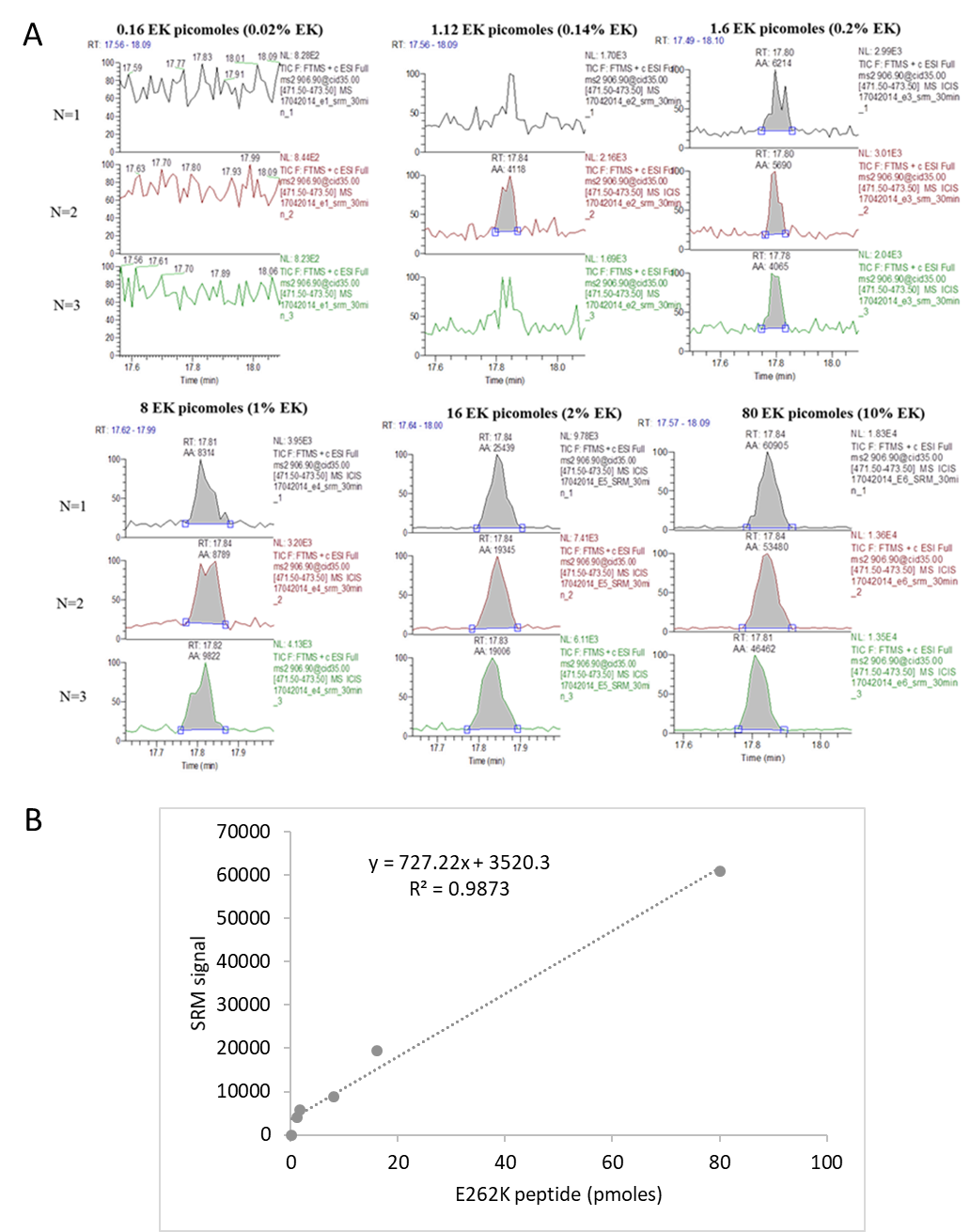
a**

**
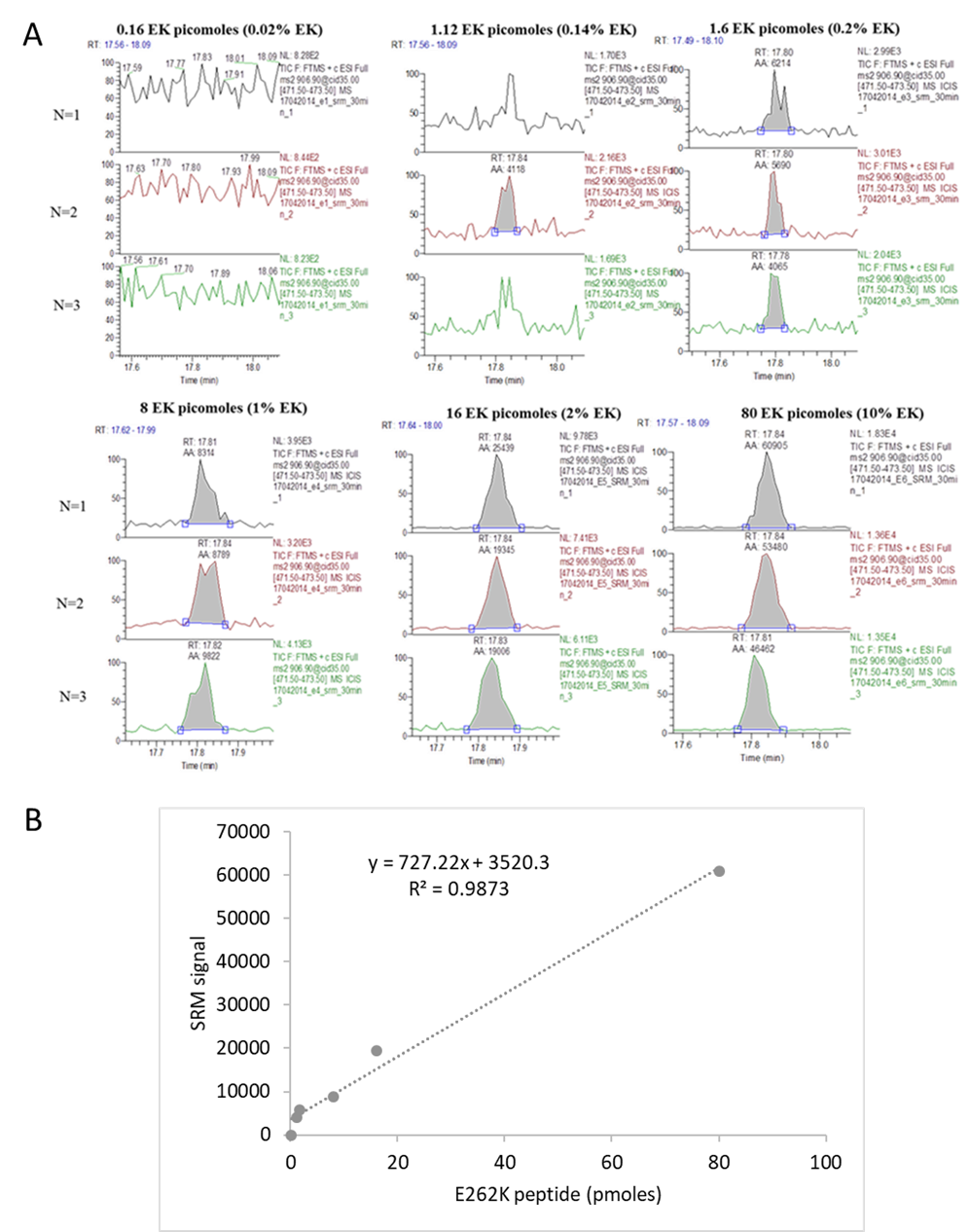
b**
